# Climate-driven changes in the predictability of seasonal precipitation

**DOI:** 10.1038/s41467-023-39463-9

**Published:** 2023-06-28

**Authors:** Phong V. V. Le, James T. Randerson, Rebecca Willett, Stephen Wright, Padhraic Smyth, Clément Guilloteau, Antonios Mamalakis, Efi Foufoula-Georgiou

**Affiliations:** 1 Environmental Sciences Division, Oak Ridge National Laboratory, Oak Ridge, TN USA; 2 Department of Civil and Environmental Engineering, University of California, Irvine, CA USA; 3 Faculty of Hydrology Meteorology and Oceanography, University of Science, Vietnam National University, Hanoi, Vietnam; 4 Department of Earth System Science, University of California, Irvine, CA USA; 5 Department of Statistics, University of Chicago, Chicago, IL USA; 6 Department of Computer Science, University of Chicago, Chicago, IL USA; 7 Computer Science Department, University of Wisconsin-Madison, Madison, WI USA; 8 Department of Computer Science, University of California, Irvine, CA USA; 9 Department of Statistics, University of California, Irvine, CA USA; 10 Department of Atmospheric Science, Colorado State University, Fort Collins, CO USA

**Keywords:** Projection and prediction, Climate and Earth system modelling

## Abstract

Climate-driven changes in precipitation amounts and their seasonal variability are expected in many continental-scale regions during the remainder of the 21st century. However, much less is known about future changes in the predictability of seasonal precipitation, an important earth system property relevant for climate adaptation. Here, on the basis of CMIP6 models that capture the present-day teleconnections between seasonal precipitation and previous-season sea surface temperature (SST), we show that climate change is expected to alter the SST-precipitation relationships and thus our ability to predict seasonal precipitation by 2100. Specifically, in the tropics, seasonal precipitation predictability from SSTs is projected to increase throughout the year, except the northern Amazonia during boreal winter. Concurrently, in the extra-tropics predictability is likely to increase in central Asia during boreal spring and winter. The altered predictability, together with enhanced interannual variability of seasonal precipitation, poses new opportunities and challenges for regional water management.

## Abstract (web summary)

This study shows that climate change will alter the sea surface temperature - precipitation relationships and our ability to predict seasonal precipitation by 2100.

## Introduction

Precipitation is a critical component of the hydrologic cycle and plays a significant role in shaping the biodiversity of terrestrial ecosystems^1,2^. It also structures socio-economic systems through influence on agricultural production^3,4^ and water resources^5^. Over the past several decades as a result of improvements in earth system models (ESMs) and the availability of high-quality climate observations, seasonal prediction of precipitation^6^ has become an increasingly important tool managers use to improve water and food security^7–10^ and support ecological restoration^11^. The predictability of precipitation at seasonal timescales is typically associated with large-scale variability of sea surface temperature (SST)^12,13^, a principal forcing driver of global atmospheric circulation. A number of studies, for example, have identified that the patterns of SST anomalies occurring over the Indian Ocean influence seasonal precipitation variability in Australia^14,15^, Africa^16–19^, and parts of Asia^20–22^. Similarly, precipitation anomalies in Amazonia are found to be associated with Atlantic and Pacific SST anomalies in the tropics^23–25^. The patterns of SST anomalies and SST-derived indices in the Pacific are also used for predicting winter precipitation over southern regions of the United States^26–29^.

Multiple lines of evidence indicate that modes of climate variability and associated teleconnections may change by the end of the 21st century^30,31^. For instance, recent studies have shown that the variability of El Niño-Southern Oscillation (ENSO)-driven precipitation is likely to be enhanced over the central-eastern Pacific owing to surface warming, even though change in the strength of ENSO-related SST variability remains uncertain^32–34^. In addition, wintertime ENSO teleconnections to Pacific North America are found to change more strongly and consistently in models where the ENSO amplitude increases with future climate warming^35,36^. Precipitation-related teleconnections of ENSO also exhibit considerable changes by the end of the 21st century in other regions, including Australia, central Africa, and the west coast of South America^30,36^. While past work has evaluated how climate modes and related teleconnections may evolve with future climate, little is known about the consequences of these changes for seasonal precipitation predictability.

In this study, we examine future changes of seasonal precipitation predictability over global land in response to climate change during the 21st century. Predictability, being an intrinsic property of a system and the theoretical upper limit to prediction, cannot itself be evaluated^37,38^; however, it is typically estimated by the predictive skill of a predictive model (what is referred to as “practical predictability”; see “Methods” section). Here, seasonal precipitation predictability at each location during each of the four seasons (boreal spring - MAM, boreal summer - JJA, boreal autumn - SON, and boreal winter - DJF) is assessed by the predictive skill of the best linear predictive model using as predictors the leading modes of global SSTs in the 3-month preceding season. We quantify changes in seasonal precipitation predictability as the difference between future (2049–2099) and historical (1964–2014) prediction skill using simulations from ESMs that contributed to the 6th Phase of the Coupled Model Intercomparison Project (CMIP6; see Methods). We provide evidence that ESMs exhibit consensus regarding future changes in the spatial patterns of seasonal precipitation predictability. These changes are likely to have significant effects on sustainable water resources management, especially in regions where limited water availability requires apportionment between agriculture and natural ecosystems (e.g., southwestern United States and southern Africa).

In our analysis, we first analyze the spatial patterns of seasonal precipitation predictability in observations and identify the main sources of predictability during the historical period. In particular, we use empirical orthogonal function (EOF) analysis to extract the principal spatial variability patterns (EOFs) and principal component time series (PCs) from global seasonal SST anomalies and apply multiple linear regression of one-season lagged precipitation anomaly series onto these PCs to develop predictive models of seasonal precipitation. We examine a combination of PCs as predictors and identify the best predictive model using the Nash-Sutcliffe Efficiency^39^ (NSE) as a skill metric (see “Methods” section). Second, we select the CMIP6 models^40^ that best capture the spatial patterns of the predictive skill in observations during the historical period and then use these models to project future changes of seasonal precipitation predictability for shared socio-economic pathways SSP2-4.5 (middle-of-the-road development) and SSP3-7.0 (regional rivalry). SSP2-4.5 represents an intermediate scenario of future emissions and is closest to the current CO_2_ emission track^41–43^. In contrast, SSP3-7.0 represents medium-to-high scenario of emissions and has a higher signal-to-noise ratio than moderate emission scenarios, making it easier to discern the climate change signal from internal variability^44^.

## Results

### Spatial patterns and drivers of predictability of seasonal precipitation

**Fig. 1:**
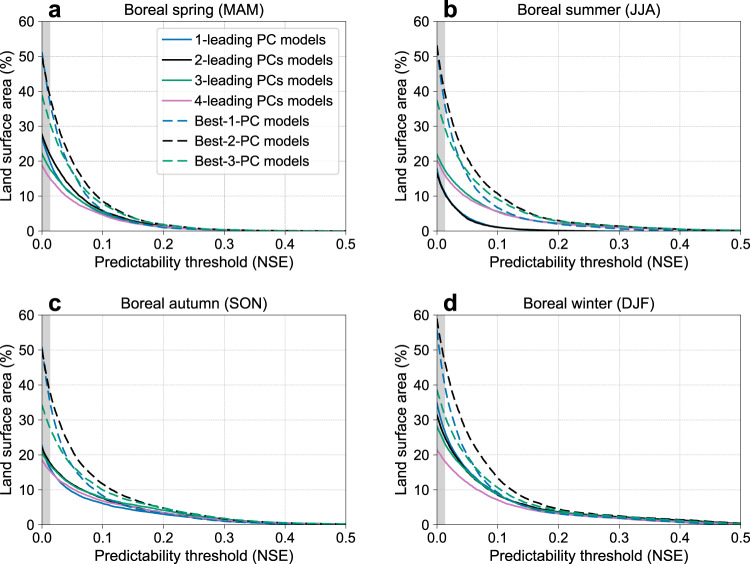
Percent of land area with predictability above a given threshold. **a**–**d** Percent of land area whose predictability, as measured by the Nash-Sutcliffe Efficiency (NSE), exceeds a given value in **a** boreal spring, **b** boreal summer, **c** boreal autumn, and **d** boreal winter, using as predictors different combinations of the first 4 principal components (PCs) of the previous season sea surface temperature (SST). These relationships were derived using an out-of-sample 5-fold cross validation approach with 1964-2014 observations of SSTs and precipitation (see “Methods” section for details). Note that the best-4-PC models are identical to the 4-leading PCs models (red solid lines) and not shown. The percent of land area is computed over all grid cells for which NSE ≥ 0 (NSE = 0 implies no predictive skill beyond climatology), excluding arid areas where the corresponding long-term mean seasonal precipitation is less than 50 mm. Grey shading indicates NSE values that are not significant at a 95% confidence level (see “Methods” section).

Seasonal precipitation predictability and the climate modes that enable this predictability are season-dependent and spatially variable. To assess predictability in the historical period, we examined different subsets of predictors selected from the first 4 PCs of observed global seasonal SST anomalies. For each subset of the predictors, the model predictive skill (measured by the NSE) was computed at each 1°×1° grid cell of the land surface, excluding arid areas whose long-term mean total seasonal precipitation was <50 mm in each three month season^45^. This low precipitation threshold was applied to avoid developing predictive models in areas with well-defined dry seasons and in desert areas where measurement uncertainties are higher. We compare in Fig. 1 the fraction of the land surface with predictability of seasonal precipitation for observations at or above a specific NSE value. Areas with NSE > 0 indicate predictive skill above using the seasonal mean climatology (i.e., climatology alone corresponds to NSE = 0). To avoid overfitting and extract the most significant predictors for each region, we examined two classes of predictive models. The first class of predictive models used the same first *n*-leading PCs (${{{n}}}{{\le }}{{{4}}}$) as the predictor(s) for multiple linear regression (called “first *n*-leading-PCs” models) in all grid cells. For this set of models, we observe an overfitting effect^46^ when more than 2 PCs are used, as revealed by a reduced NSE computed from an out-of-sample 5-fold cross validation, except during boreal summer ( JJA) where the best performance is achieved with 3 PCs. The second class of predictive models used as predictors only the subset of any of the 4 PCs of SSTs that provided the highest level of model performance for each grid cell (called “best-*n*-PCs” models, ${{{n}}}{{\le }}{{{4}}}$). For this second class of model, analysis of NSE from the cross validation reveals that the best-2-PCs model (black dashed lines in Fig. 1) is globally optimal for all seasons. The added flexibility of combining 2 different PCs for each land grid also provides considerable improvement in model performance compared to the first *n*-leading-PCs class of models (Fig. 1), and we therefore used this model as the predictive model for all subsequent analyses, i.e., for the assessment of predictability in CMIP6 models during the historical era and future periods from both the SSP2-4.5 and SSP3-7.0 scenarios.

Our analysis of the observations reveals strong seasonal variations in the degree of precipitation predictability, providing a useful benchmark for evaluating CMIP6 models. Boreal winter (DJF) precipitation is most predictable with about 58% of land surface area showing NSE > 0 and a larger fraction of land area showing predictability at or above any other NSE value, compared to precipitation in other seasons (Fig. 1). In contrast, precipitation in boreal spring (MAM) is the least predictable with about 49% of the global land area having an NSE > 0, and most of the regions showing NSE < 0.25.

**Fig. 2:**
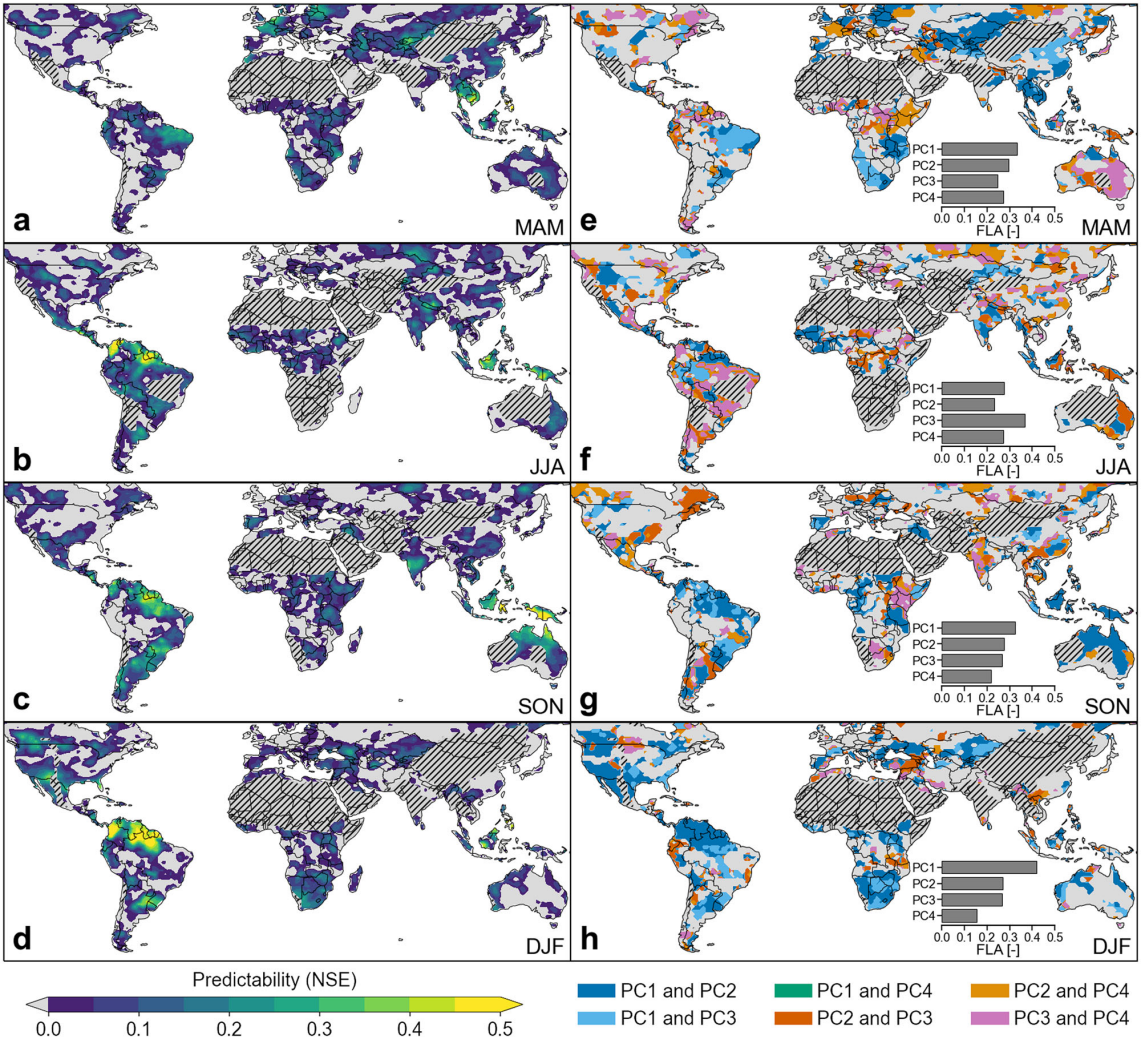
Predictability of seasonal precipitation and associated sources of predictability based on observations. **a**–**d**, Predictability of seasonal precipitation measured by the Nash-Sutcliffe Efficiency (NSE) in **a** boreal spring, **b** boreal summer, **c** boreal autumn, and **d** boreal winter obtained from the best-2-PCs model, with principal components (PCs) extracted from the previous season sea surface temperature (SST) over the 1964-2014 period using observations. **e**–**f**, Sources of predictability of seasonal precipitation (the best-2-PCs used in the predictive model) in **e** boreal spring, **f** boreal summer, **g** boreal autumn, and **h** boreal winter. Inset bar plots in **e**, **f** show the fraction of land area (FLA) associated with the PCs as sources of predictability. Grey dashed regions in **a**–**h** indicate arid areas whose long-term mean total seasonal precipitation is <50 mm, which were excluded from the analyses.

Figure 2 shows the spatial patterns of seasonal precipitation predictability and the associated PCs (predictors) used at each grid cell for the best-2-PCs model applied to the observations from the historical period. Seasonal precipitation predictability varies considerably by region and season (Fig. 2a–d). Much of the Amazonia region shows high predictability during boreal winter (Fig. 2d) that is consistent with past work identifying high levels of SST-driven predictability in the northern South America during this season^24^. Relatively high values of seasonal precipitation predictability are also found in the northwestern and southern parts of the United States during boreal winter where precipitation is known to have teleconnections with Pacific SSTs^27,28^. In contrast, northern Australia has the highest predictability during boreal autumn (Fig. 2c). Predictability across the Maritime Continent is relatively high in all seasons, with a peak in boreal autumn. This pattern is in good agreement with past work identifying that the predictability of precipitation in the Maritime Continent is higher than in most other places because of the strong and robust influence of ENSO (and its onset) near the tropical warm pool^47^. As a function of latitude, seasonal precipitation predictability is higher in the tropics than in the extra-tropics (Supplementary Fig. 1). This pattern appears consistent with the fact that tropical precipitation is more predictable than extratropical precipitation at time scales of months to a year^48,49^, and reflects the fact that tropical predictability is mostly derived from the response of moist convection to slowly varying forcing from SST^50^.

The different PCs used in the best-2-PCs model at each location are shown in Fig. 2e–h. PC1 has the spatial structure (Supplementary Fig. 2a1–d1) and year-to-year variability (Supplementary Fig. 3) of ENSO and is the single most important PC during boreal autumn, winter, and spring in the best-2-PCs model. Depending on the season, PC1 explains 18.6% (JJA) to 31.8% (SON) of the variance of global SST anomalies. Meanwhile, PC2 appears to resemble the Pacific Decadal Oscillation^51^ (PDO) pattern, which has an ENSO character but is stronger at high northern latitudes and weaker in the tropics (Supplementary Fig. 2a2–d2). PC3 and PC4 seem to be associated with the Atlantic Multidecadal Oscillation (AMO)^52^ and North Pacific Gyre Oscillation (NPGO)^53^ patterns, respectively (Supplementary Fig. 2a3–d4). Overall, the principal spatial variability patterns are relatively consistent among seasons in Supplementary Fig. 2, except PC4, suggesting that dominant teleconnections often persist across multiple seasons. These spatial patterns are also consistent with those shown in a previous study^54^. However, as the variances explained by PC2 to PC4 are comparatively close to each other, their underlying physical modes of variability are likely to be mixed. This mixing may lead to low correlations between high order PCs and corresponding climate indices in some seasons. One could use an unmixing mechanism, such as rotation of PCs^55^, to possibly increase the physical interpretability of these modes. This would however not affect the predictability results dramatically, as linearly re-combining the PCs would not change their total information content and would likely result in the same predictive power through a linear predictive model.

During boreal winter, PC1 (ENSO) contributes as a predictor of seasonal precipitation in over 42% of the global land with predictability above climatology (i.e., summing the land surface area of all 1° × 1° land grid cells with NSE > 0; see “Methods” section) and plays an even more dominant role in places with higher levels of predictability, including northeastern Amazonia, southern United States, southern Africa, and the Maritime Continent. This pattern of influence is consistent with past analyses^56–58^ evaluating the impacts of ENSO teleconnections on boreal winter precipitation. However, the dominant PC regulating predictability also varies by season. For instance, PC3 emerges as the primary driver of boreal summer precipitation for about 37% of the land surface area (see Fig. 2f).

### Future changes in seasonal precipitation predictability

#### Selection of climate models

**Fig. 3:**
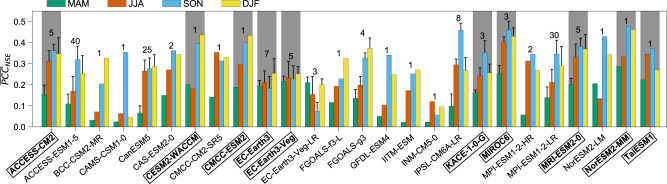
Comparison of spatial patterns of predictability scores between observations and CMIP6 models. Pattern correlation coefficients of the map of predictability score (${{{{{\rm{PC}}}}}}{{{{{{\rm{C}}}}}}}_{{{{{{\rm{NSE}}}}}}}$) between observations and 26 CMIP6 models for each season. In each model, the map of predictability score for each ensemble member is obtained from the linear model that uses the best 2 principal components (PCs) as predictors for each grid point. The bars and numbers on top represent the multi-ensemble mean (${\overline{{{{{{\rm{PCC}}}}}}}}_{{{{{{\rm{NSE}}}}}}}$) and the number of ensemble members (${n}_{e}$) of each model, respectively. For models that ${n}_{e}\ge 3$, vertical lines indicate one standard deviation of ${{{{{\rm{PC}}}}}}{{{{{{\rm{C}}}}}}}_{{{{{{\rm{NSE}}}}}}}$. Grey shading indicates the best 10 performing models (boldface, marked in boxes) that were selected for analyses of future changes.

To evaluate future changes in seasonal precipitation predictability, we first identify from 26 ESMs (including 154 ensemble members, Supplementary Table 2) the subset of ESMs which best reproduce in the historical period the spatial patterns of precipitation predictability compared to observations (Fig. 3; see “Methods” section). The selected best 10 performing CMIP6 models show that they predict well for the right reasons, i.e., they also accurately reproduce the first EOF of SST variability (Supplementary Fig. 4 shows EOF1 during winter), giving further confidence in the selection of these models. The multi-model ensemble (MME) means of the seasonal precipitation predictability (i.e., the grid-wise average NSE values across the different models and model ensembles; see “Methods” section) for the 10 selected climate models (including 32 ensemble members) demonstrate the ability of these models on average to capture the spatial and seasonal predictability patterns of the observations (Supplementary Fig. 5). Similar to the observations, the selected models show higher levels of seasonal precipitation predictability during boreal winter than other seasons (Supplementary Fig. 5d). Detailed comparisons of boreal winter precipitation predictability between each model and observations are further shown in Supplementary Fig. 6.

#### Future change

**Fig. 4:**
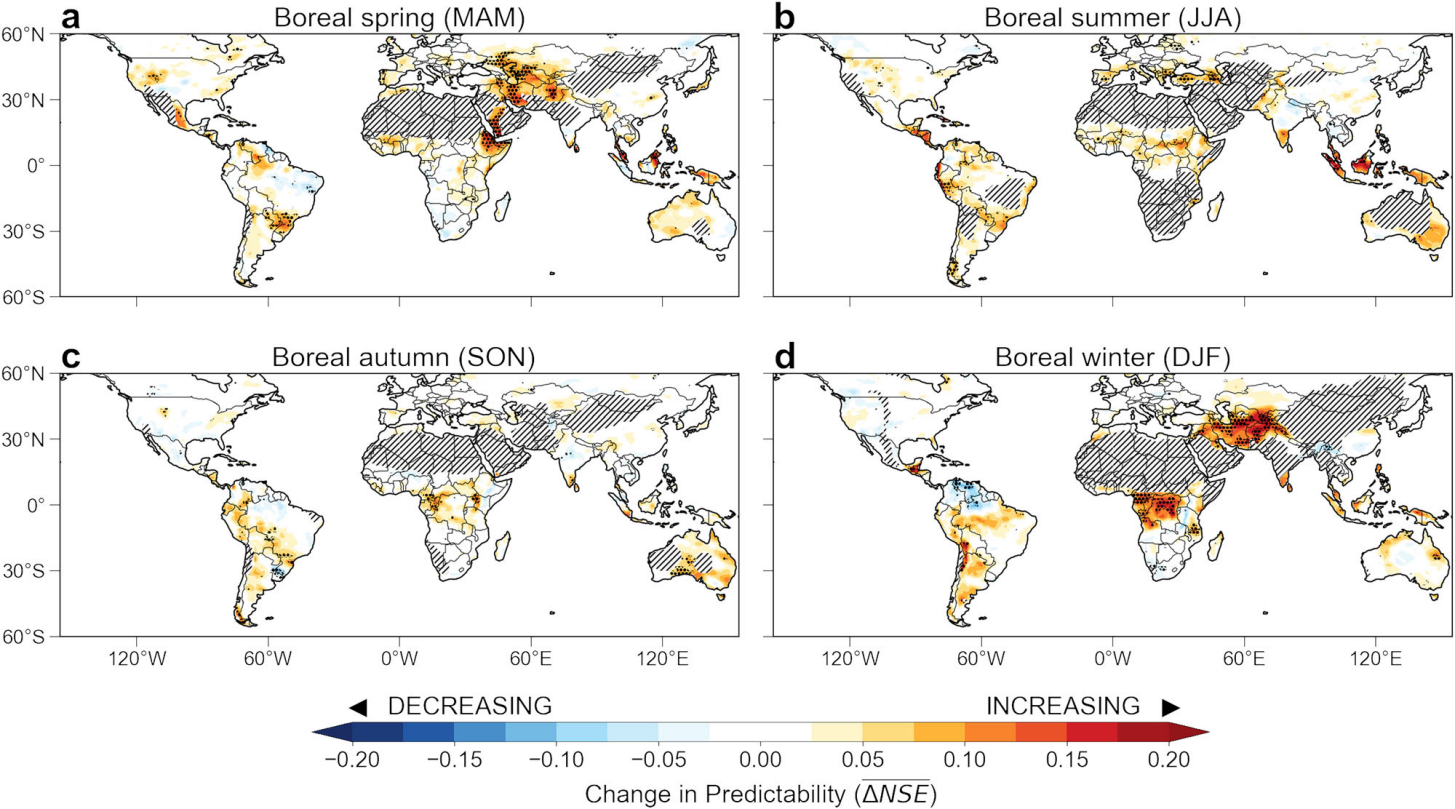
Projected changes in the predictability of seasonal precipitation under global warming scenario SSP3-7.0. **a**–**d**, Multi-model ensemble (MME) mean changes in seasonal precipitation predictability ($\overline{\varDelta {{{{{{\mathrm{NSE}}}}}}}}$) between historical (1964–2014) and future (2049–2099) periods from 32 ensemble members of the best 10 performing CMIP6 models (see Fig. 3) for **a** boreal spring, **b** boreal summer, **c** boreal autumn, and **d** boreal winter. Stippling indicates regions where at least 80% of the models show the same sign of change. Grey dashed regions indicate arid areas whose long-term mean total seasonal precipitation is <50 mm, which were excluded from the analyses.

By the end of the century for the medium-to-high emission (SSP3-7.0) scenario, the selected CMIP6 models project several consistent continental-scale changes in seasonal precipitation predictability (Fig. 4). In particular, seasonal precipitation predictability is likely to increase in the tropics between 23^o^N and 23^o^S, except for northern South America and eastern India. Models show the largest and most consistent increases of predictability in the Maritime Continent during boreal summer and central Africa during boreal winter. In the extra-tropics, seasonal precipitation predictability is projected to increase significantly in central Asia (including Iran, Afghanistan, and Turkmenistan) during boreal winter and boreal spring. The proportion of models that show an increase (${{\rm{P}}}_{\Delta {{\rm{NSE}}}}^{+}$) or decrease (${{\rm{P}}}_{\Delta {{\rm{NSE}}}}^{-}$) in predictability of more than 0.05 in NSE, representing a consensus among selected models, is further shown in Supplementary Fig. 7. In general, the CMIP6 models used in our study agree on the sign of the projected changes in predictability for a number of regions, including central Africa, central Asia, Amazonia, and Maritime Continent. For SSP2-4.5, the selected CMIP6 models project similar spatial patterns of changes in seasonal precipitation predictability (Supplementary Fig. 8) but at smaller magnitudes compared to SSP3-7.0, owing to the slower warming rate of future climate in SSP2-4.5. This consistency among models and experiments provides confidence in our finding that seasonal precipitation predictability is robust within the CMIP6 models, and this change is likely driven by global warming. While the MME mean estimate of predictability also changes in several other regions, the high level of variability among different model estimates limits our ability to draw a robust conclusion regarding the possible sign and magnitude of potential change.

We hypothesize that changing SST variability and total seasonal precipitation variability over land contribute to the alteration in seasonal precipitation predictability during the latter half of the 21st century. To investigate this hypothesis, we examined changes in SST and precipitation variability between the future (SSP3-7.0 scenario) and historical periods. For SST, depending on the season, an average of 7 of 10 selected climate models (70%) show an increase in both the total variance explained by the first PC (Supplementary Fig. 9) and the magnitude of Niño3.4 SST variability (Supplementary Fig. 10), representing a future increase in the dominance of ENSO dynamics as a driver of global interannual SST variability, with the MME mean variability explained by PC1 increasing the most in boreal autumn ($2.3\,\pm\,4.8\%$). The change in SST was further supported by a comparison between EOF1 patterns over the two periods, that is ${\overline{\Delta {{\rm{EOF1}}}}}=\left\langle {{\rm{EOF1}}}_{future}- {{\rm{EOF1}}}_{historical}\right\rangle$ where the angular brackets denote the ensemble average over the 10 selected models, showing a coherent structure, weakened over the equatorial central and eastern Pacific during boreal spring, but enhanced during boreal autumn and boreal winter (Supplementary Fig. 11). This projected amplification of ENSO may create favorable conditions for strengthening the ENSO teleconnections over many land regions driving increased interannual and seasonal variability in regional temperature and precipitation^30,59^.

In addition, most CMIP6 models project an increase in year-to-year variability of seasonal SST in the north Atlantic, tropical central Pacific, and near the Maritime Continent region (Supplementary Fig. 12). Potential consequences of increases in both the mean state and variability of tropical SSTs include an increased frequency of extreme El Niño events due to promoting atmospheric convection and an eastward shift of ENSO precipitation teleconnections^60^. For precipitation, projected changes in the seasonal precipitation amounts differ by region and season (Supplementary Fig. 13a–d). Overall, seasonal precipitation is projected to increase in the Northern Hemisphere temperate and boreal zones, but to decrease at varying degrees in Central and South Americas and southern Africa in most seasons^61–63^. However, models consistently project an increase in interannual precipitation variability in all seasons and in many regions, except a modest decrease in southern Africa and eastern South America during boreal autumn (Supplementary Fig. 13e–h). Previous studies have shown that changes in SST variability may potentially affect atmospheric teleconnection patterns^64,65^, which subsequently influence the precipitation predictability from SST. The atmosphere may also become less efficient at propagating dynamical signals from tropical climate modes into the extra tropics as a consequence of more rapid eddy expansion and dissipation from climate warming^66^. This may explain some of the structure of predictability changes we see in the northern extratropics, although future research is needed to identify interactions between this mechanism and the intensification of ENSO SST variability observed in many of the models (Supplementary Figs. S9 and 10).

From a wildfire and drought stress perspective, northern South America appears particularly vulnerable based on the results of our analysis. Specifically, precipitation during the dry season (in boreal winter) is projected to decline, interannual variability is projected to increase, and seasonal predictability is expected to decline, increasing the risk of fire ignition in tropical forest ecosystems, the likelihood that fires will grow to larger sizes^67,68^. At the same time, interannual variability is projected to increase, potentially contributing to more extreme periods of drought and fire activity. Finally, seasonal predictability is expected to decline, limiting options for seasonal fire prediction^69^ and risk mitigation.

In contrast, the increase of predictability in central and northern Africa and across the Maritime Continent could be attributed to the increase of ENSO SST variability and changes in the tropical SST-precipitation relationship, and may strengthen resilience with respect to peat and ecosystem management. Further evaluation of the mechanisms responsible for the projected changes in precipitation predictability identified here will require use of idealized model experiments^70^ or climate network analysis that can depict causality^71,72^.

We note that seasonal precipitation predictability, as defined here based on the predictive skill of the best linear model that uses as predictors previous-season SSTs, cannot be directly compared to the “potential precipitation predictability” (PPP) concept, which was reported in a recent study^70^ to decrease under global warming. PPP was defined based on the multiscale dynamics of precipitation alone and not considering changes in the sources of predictability (i.e., SSTs) or atmospheric teleconnections under warming. SST persistence or ocean memory, a major source of predictability in the climate system, is projected to increase at week-to-week^73^ but decrease at year-to-year^74^ time scales. These opposite trends in SST persistence are likely to have different effects on precipitation variability at different time scales and on the lead times at which SST predicts precipitation. While these findings have implications for changes in persistence-based predictions of sea surface thermal conditions at corresponding timescales, the effect of changes in ocean memory on the relationships between SST and (lagged) precipitation at a seasonal timescale remains unknown and is an important topic for future study. Further examination of the changes in the persistence of SST at seasonal time scale may be helpful for understanding the mechanisms responsible for future changes in seasonal precipitation predictability.

## Discussion

Climate warming is expected to modify primary climate modes of variability across scales and also their teleconnections with precipitation over land^75,76^. In this study, we examine projected future changes of seasonal precipitation predictability from previous-season SSTs caused by anthropogenic forcing of the climate system. We show that climate warming may increase the interannual variability of SSTs and precipitation leading to changes in seasonal precipitation predictability in both the tropics and extra-tropics. Precipitation predictability is projected to increase in the tropics throughout most of the year except in northern South America. In the extra-tropics, precipitation predictability is likely to increase in central Asia during wintertime.

Changes in seasonal precipitation amounts and their interannual variability under global warming pose acute challenges for regional water resources management. These challenges will be further amplified by a decrease in our ability to perform seasonal forecasts with several months lead time, especially in “hotspots” where precipitation is critical for agriculture and ecosystems or areas where base-state precipitation is very high or low. Collectively, the results from our work identify several high-risk regions and highlight the importance of understanding the causal SST-seasonal precipitation relationships in state-of-the art climate models for improving seasonal prediction of precipitation, a necessary condition for sustainable management of water resources during a period of rapid climate change.

## Methods

### Observations

Seasonal average sea surface temperature (SST) at 1° × 1° spatial resolution was derived from the COBE-SST2 datasets. Seasonal total precipitation over land was derived from two data sets: the Global Precipitation Climatology Project (GPCP; 2.5° × 2.5° resolution) and Global Precipitation Climatology Center (GPCC; 1° × 1° resolution). The GPCP integrates analyses from satellite and gauge measurements but is only available since 1979. The GPCC is based on gauge analysis only and available since 1948. We used GPCP as the main precipitation dataset and combined GPCP (bilinearly interpolated to 1° × 1° resolution) and GPCC to extract observations of seasonal total precipitation (at 1° × 1° resolution) over land from 1964 to 2014, resulting in 50 years for each season. Specifically, at each location and season, using the overlapping period 1979–2014, the differences in the mean and variability of the two datasets were evaluated. A linear adjustment was then applied to GPCC data for the period 1964–1979 to correct for the potential differences of the two datasets before combining with GPCP.

Seasonal anomalies of precipitation and SST referenced to the climatological mean of each season were constructed and detrended prior to analyses. Detrending was performed by subtracting a centered moving average from the original anomaly time series, where the centered moving average was calculated over a time window of length $\tau=11$ years. In this study, the 4 seasons were defined as: boreal spring - MAM (March–April–May); boreal summer - JJA (June–July–August); boreal autumn - SON (September–October–November); and boreal winter - DJF (December–January–February).

### Climate Models

We examined a total of 26 CMIP6 models in our analysis (including 154 ensemble members for each experiment; Supplementary Tables 1 and 2). The criteria for including a given model were whether it had simulations available for the historical (1964-2014, recent past climate) and SSP3-7.0 and SSP2-4.5 (2049-2099, future climate) experiments at the time the data were downloaded (October 2022). For each model, we used only initial condition ensemble members that are available in all experiments for consistency. For the historical experiment, all simulations were forced by common datasets using the observed greenhouse gas (GHG) concentrations and aerosol emissions^77,78^. For the SSP3-7.0 experiment, all simulations were prescribed with the same future emissions and land use changes that follow the regional rivalry SSP3 pathway with a global mean forcing of 7.0 Wm^−2^ by 2100 relative to the pre-industrial period^44,79,80^. Similarly, for SSP2-4.5, all the simulations followed the SSP2 pathway (middle of the road development) with a global mean forcing of 4.5 Wm^−2^ by 2100 relative to the pre-industrial period. All model outputs and observations were resampled to a common 1° × 1° spatial resolution by using bilinear interpolation. Similar to the observations, seasonal anomalies of each field referenced to the climatological mean of each season were constructed and detrended using the moving average method prior to all analyses. Some climate models do not reasonably capture the observed patterns of seasonal precipitation predictability (as shown in Fig. 2), and these models were excluded from the analysis for evaluating future changes of predictability (see details in Climate Model Selection for Future Projections section). We also repeated the analyses using linearly detrended anomalies, which yield similar results to the moving average detrending method (See Supplementary Figs. 14 and 15), although the changes of seasonal precipitation predictability were found to be slightly smaller.

### Empirical orthogonal function analysis of SSTs

We performed an empirical orthogonal function (EOF) analysis^81^ of global seasonal SSTs to: (i) isolate dominant spatial modes of variability (EOFs) and (ii) reduce the precipitation predictors to only a smaller number of principal component (PC) time series. The spatial patterns of EOFs and the percent variance explained as extracted from the observations of SST are presented in Supplementary Fig. 2. The leading four PCs, which collectively explain at least 40% of the global seasonal SST variance, were retained for predicting seasonal precipitation in the next season. For each CMIP6 model, EOF analysis was applied independently across experiments for the historical and future periods.

### Assessment of predictability

To examine seasonal precipitation predictability, we constructed the best multiple linear regression model between seasonal precipitation and the leading modes of global SSTs in the previous season. Specifically, the predictands $({\hat{{{{{{\boldsymbol{y}}}}}}}}^{{{{{{\mathscr{l}}}}}}{{{{{\mathscr{,}}}}}}s})$ were seasonal precipitation anomalies in season $s$ (e.g., boreal winter) at each grid point ${{{{{\mathcal{l}}}}}}$ of the land surface, and the predictors (${{{{{{\boldsymbol{x}}}}}}}_{j}^{s-1}$) were the PC time series obtained from EOF analysis of global SST anomalies in the previous season $s-1$ (e.g., boreal autumn):

1 $${\hat{{{{{{\boldsymbol{y}}}}}}}}^{{{{{{\mathscr{l}}}}}}{{{{{\mathscr{,}}}}}}s}={\beta }_{0}^{{{{{{\mathscr{l}}}}}}}+\mathop{\sum}\limits_{j}{\beta }_{j}^{{{{{{\mathscr{l}}}}}}}\times {{{{{{\boldsymbol{x}}}}}}}_{j}^{s-1}$$

where the subscript $j$ indicates the indices of the best 2 predictors identified for each grid point (see Fig. 1 and Predictability Score section below). At each land grid point ${{{{{\mathcal{l}}}}}}$ the combination of any 2 PCs chosen from the first 4 PCs showing the highest predictive skill was selected as the corresponding predictors of precipitation for the grid point. The parameters to fit were the intercept ${\beta }_{0}^{{{{{{\mathscr{l}}}}}}}$ and the coefficient ${\beta }_{j}^{{{{{{\mathscr{l}}}}}}}$ associated with each predictor ${{{{{{\boldsymbol{x}}}}}}}_{j}^{s-1}$, which were estimated from the in-sample data by means of a 5-fold cross validation to mitigate overfitting^46^ and ensure a rigorous assessment. The regression model was then evaluated using the remaining out-of-sample data. Overall, the best-2-PCs predictor model exhibited the highest predictability score compared to other combinations of PCs. These best 2 predictors (i.e., PCs) were identified as the two primary sources of predictability at each grid cell and Fig. 2e–h (inset bar plots) show the fraction of land area (FLA; latitude-dependence adjusted) whose predictability is derived from those PCs, calculated as:

2 $${{{{{{\rm{FLA}}}}}}}_{j,s}=\frac{{\sum }_{{{{{{\mathscr{l}}}}}}{{{{{\mathscr{=}}}}}}1}^{{N}_{j,s}}{{{{{\rm{A}}}}}}{{\cos }}({{{{{{\rm{\lambda }}}}}}}_{{{{{{\mathscr{l}}}}}}})}{{\sum }_{{{{{{\mathscr{l}}}}}}{{{{{\mathscr{=}}}}}}1}^{{N}_{G}}{{{{{\rm{A}}}}}}{{\cos }}({{{{{{\rm{\lambda }}}}}}}_{{{{{{\mathscr{l}}}}}}})}$$

where ${{{{{\rm{A}}}}}}$ is the area of a land grid cell on a uniform latitude $\times$ longitude grid; ${{{{{{\rm{\lambda }}}}}}}_{{{{{{\mathscr{l}}}}}}}$ is the latitude of the land grid cell ${{{{{\mathcal{l}}}}}}$; ${N}_{G}$ is the number of land grid cells over the entire globe, excluding arid regions; and ${N}_{j,s}$ is the number of land grid cells for which ${{{{{{\rm{PC}}}}}}}_{j}$ contributes as one of the two predictors of precipitation for the season $s$. Since two PCs serve as sources of predictability for a land grid cell for a season $s$, we note that $\mathop{\sum }\nolimits_{j=1}^{4}{{{{{{\rm{FLA}}}}}}}_{j,s}\in [0,2]$.

For each CMIP6 model, the above analysis was repeated independently for each ensemble member to get the model average (and uncertainty if multiple ensemble members were available), and the best-2-predictor model was applied independently across experiments for the historical and future periods.

### Predictability score

The Nash-Sutcliffe Efficiency (NSE) was used to evaluate the predictive skill of each fitted model and served as a measure of predictability (predictability score). For a particular season and at a specific grid point on the land surface, the NSE is defined as:

3 $${{{{{\rm{NSE}}}}}}=1-\frac{{\sum }_{i=1}^{n}{\left({y}_{i}-{\hat{y}}_{i}\right)}^{2}}{{\sum }_{i=1}^{n}{\left({y}_{i}-\bar{y}\right)}^{2}}$$

where ${y}_{i}$ is seasonal precipitation anomaly for the ${i}{{{\mathrm{th}}}}$ year; ${\hat{y}}_{i}$ is the predicted seasonal precipitation anomaly for the ${i}{{{\mathrm{th}}}}$ year; $\bar{y}$ is the long-term average of seasonal total precipitation anomaly (equal to 0 in this case); and $n$ is the number of years. NSE < 0 means that the model has no predictive ability (worse than climatology for which NSE = 0). NSE > 0 indicates an acceptable level of model predictive performance, with NSE = 1 indicating a perfect prediction.

To estimate the 95% confidence level that NSE is not significant shown in Fig. 1, we used the Monte Carlo simulation approach as follows: (i) for each land grid cell (excluding arid regions) and each season, observed seasonal precipitation anomaly time series was randomly shuffled and linearly regressed against the original time series of the two corresponding predictors (i.e., PCs) using a 5-fold cross validation to calculate the NSE values; (ii) repeated the process 1000 times (all land grid cells and 4 seasons for each time) and identified the 95th percentile of the obtained NSE distribution is ~0.013.

For a climate model $m$ with ${n}_{e}$ ensemble members, the multi-ensemble mean (i.e., the average across all ${n}_{e}$ ensemble members of that model $m$) of change in seasonal precipitation predictability for a particular season and for each grid cell is computed as:

4 $$\Delta {{{{{\rm{NS}}}}}}{{{{{{\rm{E}}}}}}}_{m}=\frac{1}{{n}_{e}}\times \mathop{\sum }\limits_{i=1}^{{n}_{e}}\left[{{\max }}\left({{{{{\rm{NS}}}}}}{{{{{{\rm{E}}}}}}}_{m,i}^{{{{{{\rm{future}}}}}}},\,0\right)-{{\max }}\left({{{{{\rm{NS}}}}}}{{{{{{\rm{E}}}}}}}_{m,i}^{{{{{{\rm{historical}}}}}}},\,0\right)\right]$$

The above equation implies that the climatology prediction is used if the best 2-PC linear model is worse than climatology. The multi-model ensemble (MME) mean (i.e., the average obtained from all selected climate models) of the change of predictability is calculated as:

5 $${\overline{\Delta {{{{{\rm{NSE}}}}}}}}=\frac{1}{M}\times \mathop{\sum }\limits_{m=1}^{M}\Delta {{{{{\rm{NS}}}}}}{{{{{{\rm{E}}}}}}}_{m}$$

in which M is the number of CMIP6 models selected from the performance evaluation (*M* = 10). Note that, despite differences in the number of ensemble members ${n}_{e}$ across models, selected climate models were given equal weight in the calculation of $\overline{\varDelta {{{{{\rm{NSE}}}}}}}$. The proportion of models showing an increase in seasonal precipitation predictability, or ${{{{{{\rm{P}}}}}}}_{\Delta {{{{{\rm{NSE}}}}}}}^{+}$ (%), is then calculated as:

6 $${{{{{{\rm{P}}}}}}}_{\Delta {{{{{\rm{NSE}}}}}}}^{+}=\frac{100}{M}\times \mathop{\sum }\limits_{m=1}^{M}\Theta \left(\Delta {{{{{{\rm{NSE}}}}}}}_{m}-\delta \right)$$

and the proportion of models showing significant decrease in predictability, or ${{{{{{\rm{P}}}}}}}_{\Delta {{{{{\rm{NSE}}}}}}}^{-}$ (%), is:

7 $${{{{{{\rm{P}}}}}}}_{\Delta {{{{{\rm{NSE}}}}}}}^{-}=\frac{100}{M}\times \mathop{\sum }\limits_{m=1}^{M}\Theta \left(-\Delta {{{{{{\rm{NSE}}}}}}}_{m}-\delta \right)$$

where $\delta$=0.05 is the threshold of significant change, $\Theta (x)$ is the Heaviside theta-function which takes the values $\Theta \left(x\right)=1$ for $x\ge 0$ and $\Theta \left(x\right)=0$ for $x\, < \,0$.

### Selection of Climate Models for Future Projection

To select climate models for assessing future changes in seasonal precipitation predictability, we examined the ability of each model in the historical period to reproduce the seasonal precipitation predictability in the observations. In particular, for a model $m$ with ${n}_{e}$ ensemble members, we calculated the multi-ensemble mean (the average of all ensemble members of that model $m$) pattern correlation coefficient (PCC) of seasonal precipitation predictability scores (represented by the spatial patterns of NSE) for each season between the observed data and historical model simulations, defined as:

8 $${\overline{{{{{{\rm{PCC}}}}}}}}_{m,{{{{{\rm{NSE}}}}}}}=\frac{1}{{n}_{e}}\times \mathop{\sum }\limits_{i=1}^{{n}_{e}}\frac{{\sum }_{{{{{{\mathscr{l}}}}}}{{{{{\mathscr{=}}}}}}1}^{N}\left({{{{{{\rm{NSE}}}}}}}_{{{{{{\rm{o}}}}}},{{{{{\mathscr{l}}}}}}}-{\overline{{{{{{\rm{NSE}}}}}}}}_{{{{{{\rm{o}}}}}}}\right)\left({{{{{{\rm{NSE}}}}}}}_{m{{{{{\mathscr{,}}}}}}{{{{{\mathscr{l}}}}}}}^{i}-{\overline{{{{{{\rm{NSE}}}}}}}}_{m}^{i}\right)}{\sqrt{{\sum }_{{{{{{\mathscr{l}}}}}}{{{{{\mathscr{=}}}}}}1}^{N}{\left({{{{{{\rm{NSE}}}}}}}_{{{{{{\rm{o}}}}}},{{{{{\mathscr{l}}}}}}}-{\overline{{{{{{\rm{NSE}}}}}}}}_{{{{{{\rm{o}}}}}}}\right)}^{2}}\sqrt{{\sum }_{{{{{{\mathscr{l}}}}}}{{{{{\mathscr{=}}}}}}1}^{N}{\left({{{{{{\rm{NSE}}}}}}}_{m{{{{{\mathscr{,}}}}}}{{{{{\mathscr{l}}}}}}}^{i}-{\overline{{{{{{\rm{NSE}}}}}}}}_{m}^{i}\right)}^{2}}}$$

where ${{{{{{\rm{NSE}}}}}}}_{{{{{{\rm{o}}}}}},{{{{{\mathscr{l}}}}}}}$ and ${{{{{{\rm{NSE}}}}}}}_{{{{{{\rm{m}}}}}},{{{{{\mathscr{l}}}}}}}^{i}$ are the predictability scores of precipitation at the same land grid point ${{{{{\mathcal{l}}}}}}$ obtained from observations and model ensemble member $i$, respectively; ${\overline{{{{{{\rm{NSE}}}}}}}}_{o}=\frac{{\sum }_{{{{{{\mathscr{l}}}}}}{{{{{\mathscr{=}}}}}}1}^{N}{{{{{{\rm{NSE}}}}}}}_{o{{{{{\mathscr{,}}}}}}{{{{{\mathscr{l}}}}}}}}{N}$ and ${\overline{{{{{{\rm{NSE}}}}}}}}_{m}^{i}=\frac{{\sum }_{{{{{{\mathscr{l}}}}}}{{{{{\mathscr{=}}}}}}1}^{N}{{{{{{\rm{NSE}}}}}}}_{m{{{{{\mathscr{,}}}}}}{{{{{\mathscr{l}}}}}}}^{i}}{N}$; and $N$ is the total number of land grid points with acceptable predictability in the observations (${{{{{{\rm{NSE}}}}}}}_{{{{{{\rm{o}}}}}}{{{{{\mathscr{,}}}}}}{{{{{\mathscr{l}}}}}}}$ > 0). This means we ignored areas with no predictive ability in the observations, meaning that the calculation of ${\overline{{{{{{\rm{PCC}}}}}}}}_{m,{{{{{\rm{NSE}}}}}}}$ only includes land grid points ${{{{{\mathcal{l}}}}}}$ where ${{{{{{\rm{NSE}}}}}}}_{{{{{{\rm{o}}}}}},{{{{{\mathscr{l}}}}}}} > 0$. For each climate model, we identified the season with the worst performance (smallest ${\overline{{{{{{\rm{PCC}}}}}}}}_{m,{{{{{\rm{NSE}}}}}}}$ among all 4 seasons):

9 $${\overline{{{{{{\rm{PCC}}}}}}}}_{m,{{{{{\rm{NSE}}}}}}}^{\ast}={\min}({\overline{{{{{{\rm{PCC}}}}}}}}_{m,{{{{{\rm{NSE}}}}}}}^{{{{{{\rm{MAM}}}}}}},{\overline{{{{{{\rm{PCC}}}}}}}}_{m,{{{{{\rm{NSE}}}}}}}^{{{{{{\rm{JJA}}}}}}},{\overline{{{{{{\rm{PCC}}}}}}}}_{m,{{{{{\rm{NSE}}}}}}}^{{{{{{\rm{SON}}}}}}},{\overline{{{{{{\rm{PCC}}}}}}}}_{m,{{{{{\rm{NSE}}}}}}}^{{{{{{\rm{DJF}}}}}}})$$

We ranked models according to their $\overline{{{{{{\rm{PCC}}}}}}}{{\,}^{\ast}}_{\!\!\!m,{{{{{\rm{NSE}}}}}}}$ (from largest to smallest) and selected the top 10 best performing models, ensuring good performance of these models throughout the year, to assess the future changes of seasonal precipitation predictability. In Fig. 3, for models that have ${n}_{e}\ge 3$ the vertical lines indicate the ensemble-to-ensemble (or internal) variability (as measured by the standard deviation of the ${{{{{\rm{PC}}}}}}{{{{{{\rm{C}}}}}}}_{m,{{{{{\rm{NSE}}}}}}}$) that arises from complex interactions among different components of each specific model. The internal variability is relatively small in the selected climate models, providing more confidence in the selection of those models for future projection. Internal variability for models that have ${n}_{e}\, < \,3$ is not shown as not enough realizations to compute a standard deviation.

### Changes in precipitation and EOFs

The differences in seasonal total precipitation and EOFs between the historical and SSP3-7.0 (or SSP2-4.5) runs defined the future changes in seasonal total precipitation and EOFs, respectively.

### Changes in Niño3.4 SST variability

Detrended time series of seasonal SST anomalies were averaged over the region of Niño3.4 (5^o^S–5^o^N, 170^o^W–120^o^W) for each season. In Supplementary Fig. 10, we compared the standard deviation of the Niño3.4 averaged SST anomalies between the historical (1964–2014) and future (2049–2099; SSP3-7.0 scenario) periods.

## Supplementary information

Supplementary Information

## Data Availability

The CMIP6 data are available at https://esgf-node.llnl.gov/search/cmip6/. COBE-SST2 data is available at https://www.esrl.noaa.gov/psd/data/gridded/data.cobe2.html. GPCP is available at https://psl.noaa.gov/data/gridded/data.gpcp.html. GPCC data is available at https://climatedataguide.ucar.edu/climate-data/gpcc-global-precipitation-climatology-centre.

## Code availability

The code associated with this paper are freely available for download in SPP repository, 10.5281/zenodo.8015084.
