# Author Correction: Climate-driven changes in the predictability of seasonal precipitation

**DOI:** 10.1038/s41467-023-39975-4

**Published:** 2023-07-18

**Authors:** Phong V. V. Le, James T. Randerson, Rebecca Willett, Stephen Wright, Padhraic Smyth, Clément Guilloteau, Antonios Mamalakis, Efi Foufoula-Georgiou

**Affiliations:** 1 Environmental Sciences Division, Oak Ridge National Laboratory, Oak Ridge, TN USA; 2 Department of Civil and Environmental Engineering, University of California, Irvine, CA USA; 3 Faculty of Hydrology Meteorology and Oceanography, University of Science, Vietnam National University, Hanoi, Vietnam; 4 Department of Earth System Science, University of California, Irvine, CA USA; 5 Department of Statistics, University of Chicago, Chicago, IL USA; 6 Department of Computer Science, University of Chicago, Chicago, IL USA; 7 Computer Science Department, University of Wisconsin-Madison, Madison, WI USA; 8 Department of Computer Science, University of California, Irvine, CA USA; 9 Department of Statistics, University of California, Irvine, CA USA; 10 Department of Atmospheric Science, Colorado State University, Fort Collins, CO USA

**Keywords:** Projection and prediction, Climate and Earth system modelling

Correction to: *Nature Communications* 10.1038/s41467-023-39463-9, published online 28 June 2023

The original version of this Article contained an error in Fig. 1, in which the vertical axes in all panels showed the cumulative distribution function (“CDF”), i.e., the fraction of land surface area with Nash-Sutcliffe Efficiency (NSE) below a given threshold. Instead, they were meant to show the percentage of land surface area with NSE above a given threshold (“Land surface area (%)”) as described in the title and caption of the Figure. This has been corrected in both the PDF and HTML versions of the Article.

