# Supplementary material for: Climate-driven changes in the predictability of seasonal precipitation
Source: Nat Commun. 2023 Jun 28;14:3822. doi: 10.1038/s41467-023-39463-9 (PMC10307878; doi:10.1038/s41467-023-39463-9)
Supplement: Supplementary file 1 — Supplementary Information [file 41467_2023_39463_MOESM1_ESM.pdf]

Supplementary Information

Climate-driven changes in the predictability of seasonal precipitation

Phong V.V. Le[1,2,3]*, James T. Randerson[2,4], Rebecca Willett[5,6], Stephen Wright[7], Padhraic Smyth[8,9], Clément Guilloteau[2], Antonios Mamalakis[10] and Efi Foufoula-Georgiou[2,4*]

[1]Environmental Sciences Division, Oak Ridge National Laboratory, Oak Ridge, TN, USA
[2]Department of Civil and Environmental Engineering, University of California, Irvine, CA, USA
[3]Faculty of Hydrology Meteorology and Oceanography, University of Science, Vietnam National University, Hanoi, Vietnam
[4]Department of Earth System Science, University of California, Irvine, CA, USA
[5]Department of Statistics, University of Chicago, Chicago, IL, USA
[6]Department of Computer Science, University of Chicago, Chicago, IL, USA
[7]Computer Science Department, University of Wisconsin-Madison, Madison, WI, USA
[8]Department of Computer Science, University of California, Irvine, CA, USA.
[9]Department of Statistics, University of California, Irvine, CA, USA
[10]Department of Atmospheric Science, Colorado State University, Fort Collins, CO, USA

Corresponding Authors

Phong V.V. Le; email: lepv@ornl.gov

Efi Foufoula-Georgiou; email: efi@uci.edu

This PDF file includes:

Supplementary Tables 1 to 2

Supplementary Figures 1 to 15

Supplementary Table 1 | Information of CMIP6 models used in this study. Names of climate models, the associated institutions and countries, and grid resolutions.

	CMIP6 Model	Institute, Country	Spatial resolution (lon × lat)	
			Atmosphere	Ocean
1	ACCESS-CM2[1]	CSIRO, Australia	1.87° × 1.25°	1.0° × 0.6°
2	ACCESS-ESM1-5[2]		1.87° × 1.25°	1.0° × 0.6°
3	BCC-CSM2-MR[3]	BCC, China	1.12° × 1.12°	1.0° × 0.78°
4	CAMS-CSM1-0[4]	CAMS, China	1.12° × 1.12°	1.0° × 0.9°
5	CanESM5[5]	CCCma, Canada	2.5° × 2.5°	1.0° × 0.6°
6	CAS-ESM2-0[6]	CAS, China	1.4° × 1.4°	1.0° × 0.9°
7	CESM2-WACCM[7]	NCAR, USA	1.25° × 0.9°	1.12° × 0.47°
8	CMCC-CM2-SR5[8]	CMCC, Italy	1.25° × 0.9°	1.0° × 0.6°
9	CMCC-ESM2[9]		1.25° × 0.9°	1.0° × 0.6°
10	EC-Earth3[10]	Europe-wide Consortium	0.7° × 0.7°	1.0° × 0.6°
11	EC-Earth3-Veg[10]		0.7° × 0.7°	1.0° × 0.6°
12	EC-Earth3-Veg-LR[10]		1.12° × 1.12°	1.0° × 0.6°
13	FGOALS-f3-L[11]	CAS, China	1.0° × 1.0°	1.0° × 0.8°
14	FGOALS-g3[12]		2.0° × 2.25°	1.0° × 0.8°
15	GFDL-ESM4[13]	NOAA-GFDL, USA	1.0° × 1.0°	0.5° × 0.3°
16	IITM-ESM[14]	CCCR-IITM, India	1.87° × 1.91°	1.0° × 0.9°
17	INM-CM5-0[15]	INM, Russia	2.0° × 1.5°	0.5° × 0.25°
18	IPSL-CM6A-LR[16]	IPSL, France	2.5° × 1.25°	1.0° × 0.54°
19	KACE-1-0-G[17]	NIMS-KMA, Korea	1.87° × 1.25°	1.0° × 0.9°
20	MIROC6[18]	JAMSTEC, Japan	1.4° × 1.4°	1.0° × 0.70°
21	MPI-ESM1-2-HR[19]	MPI-M, Germany	0.94° × 0.94°	0.45° × 0.45°
22	MPI-ESM1-2-LR[20]		1.87° × 1.87°	1.4° × 0.82°
23	MRI-ESM2-0[21]	MRI, Japan	1.12° × 1.12°	1.0° × 0.5°
24	NorESM2-LM[22]	NCC, Norway	2.5° × 1.87°	1.0° × 0.47°
25	NorESM2-MM[22]		1.25° × 0.94°	1.0° × 0.47°
26	TaiESM1[23]	AS-RCEC, Taiwan	0.9° × 1.25°	1.12° × 0.47°

Supplementary Table 2 | Pattern correlation coefficients of the maps of predictability score ($\mathrm{PCC_{NSE}}$) between CMIP6 models and observations for each season during historical (1964-2014) period. In models that $n_e \geq 3$, the standard deviations represent model uncertainty in each season. The best 10 performing models selected to assess future changes in predictability are in grey shading.

	CMIP6 Models	Number of ensembles n_e	$\overline{\mathrm{PCC}}_{\mathrm{NSE}}$			
			MAM	JJA	SON	DJF
1	ACCESS-CM2	5	0.16±0.04	0.31±0.05	0.36±0.03	0.35±0.08
2	ACCESS-ESM1-5	40	0.11±0.05	0.17±0.07	0.32±0.06	0.25±0.08
3	BCC-CSM2-MR	1	0.03	0.07	0.20	0.33
4	CAMS-CSM1-0	1	0.02	0.06	0.35	0.05
5	CanESM5	25	0.07±0.03	0.27±0.05	0.28±0.05	0.29±0.06
6	CAS-ESM2-0	2	0.15	0.27	0.36	0.34
7	CESM2-WACCM	1	0.20	0.18	0.39	0.43
8	CMCC-CM2-SR5	1	0.14	0.35	0.31	0.33
9	CMCC-ESM2	1	0.19	0.30	0.40	0.43
10	EC-Earth3	7	0.19±0.03	0.21±0.05	0.18±0.06	0.25±0.07
11	EC-Earth3-Veg	5	0.22±0.02	0.24±0.08	0.23±0.06	0.25±0.03
12	EC-Earth3-Veg-LR	3	0.21±0.03	0.15±0.09	0.07±0.04	0.20±0.03
13	FGOALS-f3-L	1	0.12	0.19	0.23	0.33
14	FGOALS-g3	4	0.14±0.04	0.20±0.04	0.33±0.02	0.37±0.05
15	GFDL-ESM4	1	0.05	0.10	0.34	0.25
16	IITM-ESM	1	0.02	0.17	0.25	0.27
17	INM-CM5-0	1	0.02	0.12	0.06	0.10
18	IPSL-CM6A-LR	8	0.10±0.06	0.29±0.03	0.46±0.03	0.27±0.06
19	KACE-1-0-G	3	0.16±0.01	0.24±0.04	0.35±0.06	0.26±0.04
20	MIROC6	3	0.25±0.04	0.40±0.02	0.46±0.04	0.43±0.04
21	MPI-ESM1-2-HR	2	0.06	0.31	0.34	0.27
22	MPI-ESM1-2-LR	30	0.14±0.05	0.21±0.06	0.35±0.07	0.29±0.09
23	MRI-ESM2-0	5	0.20±0.03	0.33±0.06	0.38±0.04	0.37±0.07
24	NorESM2-LM	1	0.21	0.13	0.43	0.34
25	NorESM2-MM	1	0.29	0.34	0.48	0.46
26	TaiESM1	1	0.22	0.35	0.37	0.27

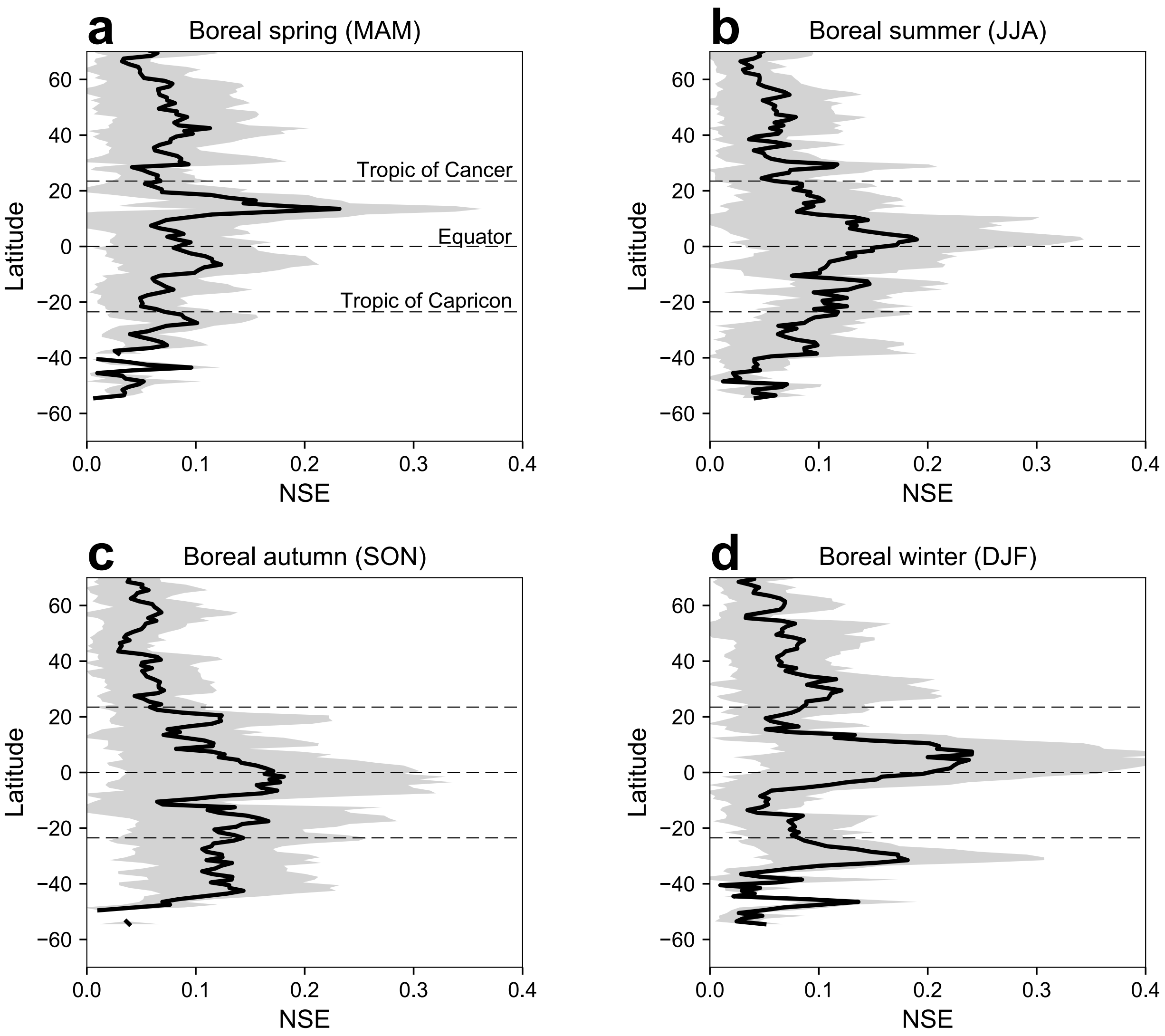

Supplementary Figure 1 | Latitudinal averages of seasonal precipitation predictability in the observations during 1964-2014. a-d, Latitudinal averages of predictability measured by the Nash-Sutcliffe Efficiency (NSE) in observations for (**a**) boreal spring, (**b**) boreal summer, (**c**) boreal autumn, and (**d**) boreal winter with shading indicating ± 1 standard deviation of predictability for each latitude across all longitudes, excluding arid areas defined in the main text.

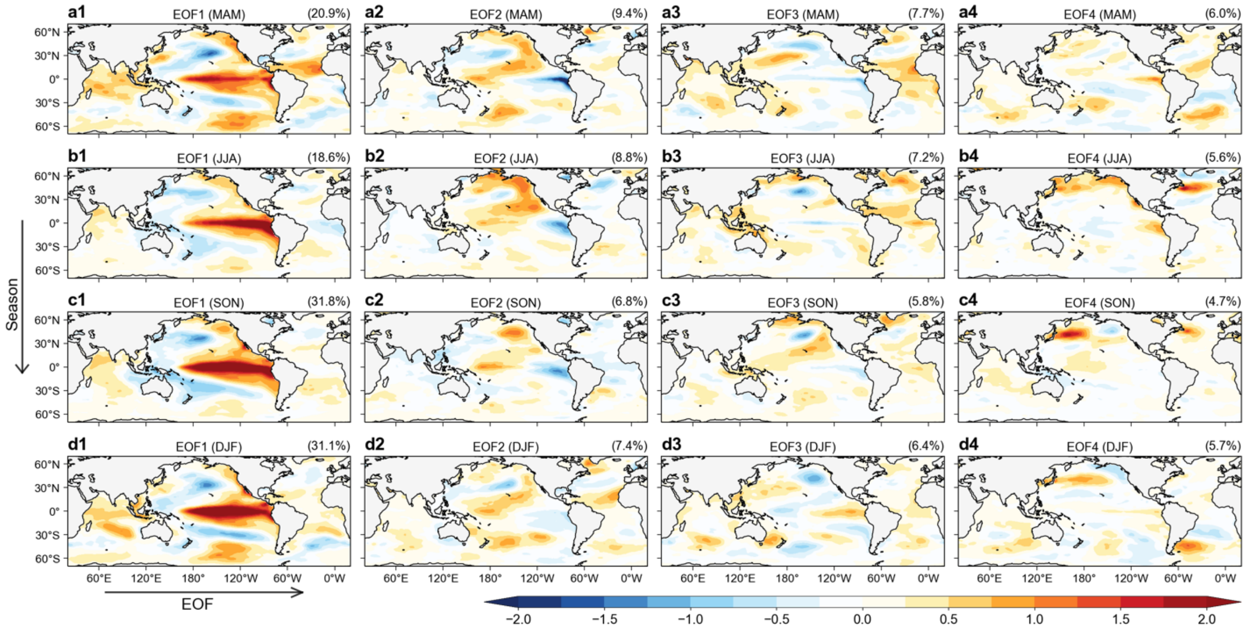

Supplementary Figure 2 | Principal variability patterns of observed sea surface temperature (SST). a-d, Four leading modes of SST variability obtained by applying an empirical orthogonal function (EOF) analysis to observed seasonal average SST anomalies in (**a1-a4**) boreal spring, (**b1-b4**) boreal summer, (**c1-c4**) boreal autumn, and (**d1-d4**) boreal winter over the historical (1964-2014) period. Numbers in parentheses on the top-right corner show the variance explained by the corresponding mode. The EOF modes are scaled by the standard deviation of the respective principal components (PCs).

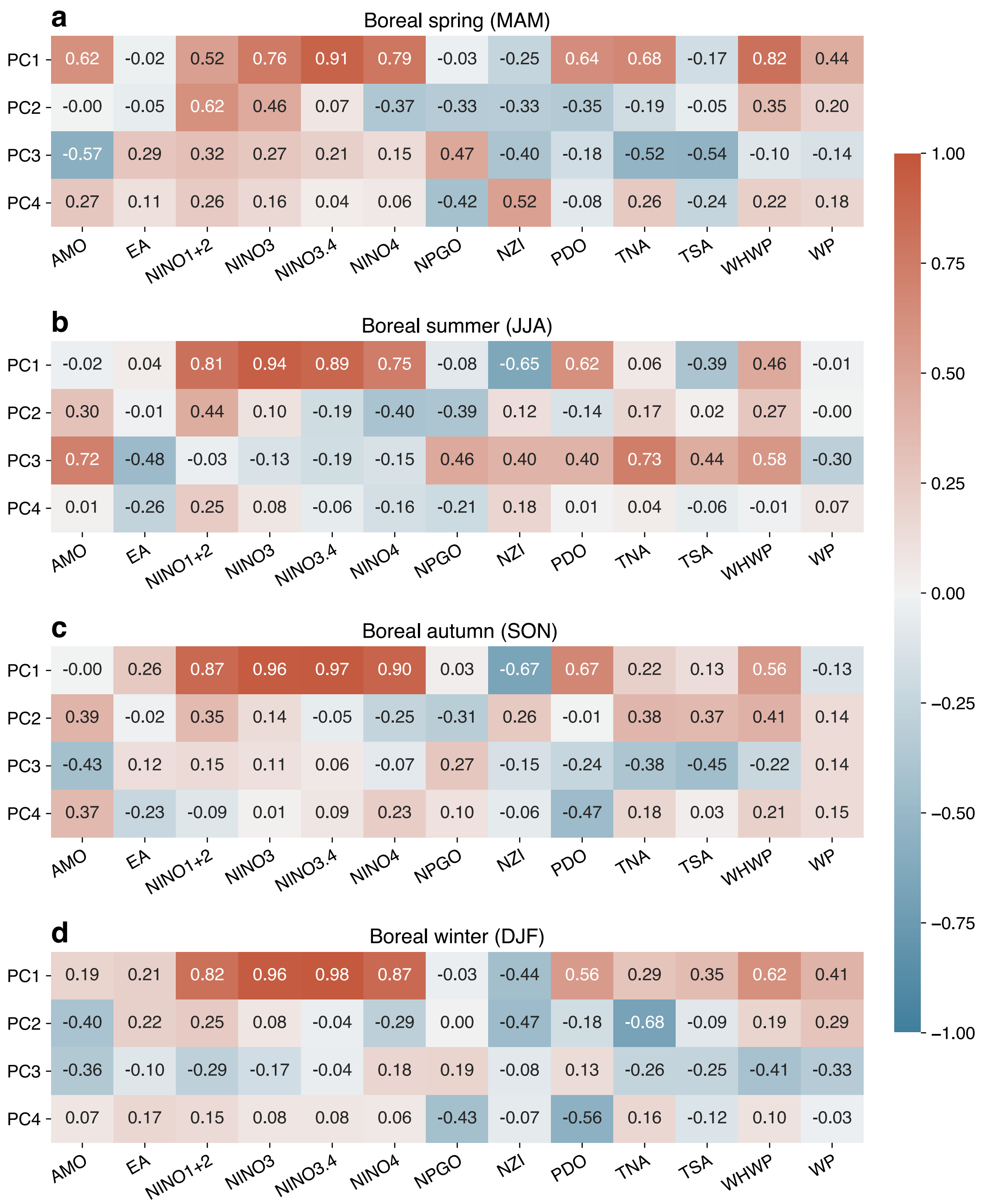

Supplementary Figure 3 | Correlation coefficients between the 4 leading principal components (PCs) and 12 climate indices (CIs) in (**a**) boreal spring, (**b**) boreal summer, (**c**) boreal autumn, and (**d**) boreal winter for observations over 1964-2014. The 12 CIs include Atlantic Multidecadal Oscillation (AMO), Eastern Asia (EA), Niño1+2, Niño3, Niño3.4, Niño4, North Pacific Gyre Oscillation (NPGO), New Zealand index (NZI), Pacific Decadal Oscillation (PDO), Tropical Northern Atlantic (TNA), Tropical Southern Atlantic (TSA), Western Hemisphere Warm Pool (WHWP), and Western Pacific (WP). High correlation between PC1 and Niño indices demonstrates that PC1 exhibits the year-to-year variability of El Niño-Southern Oscillation (ENSO).

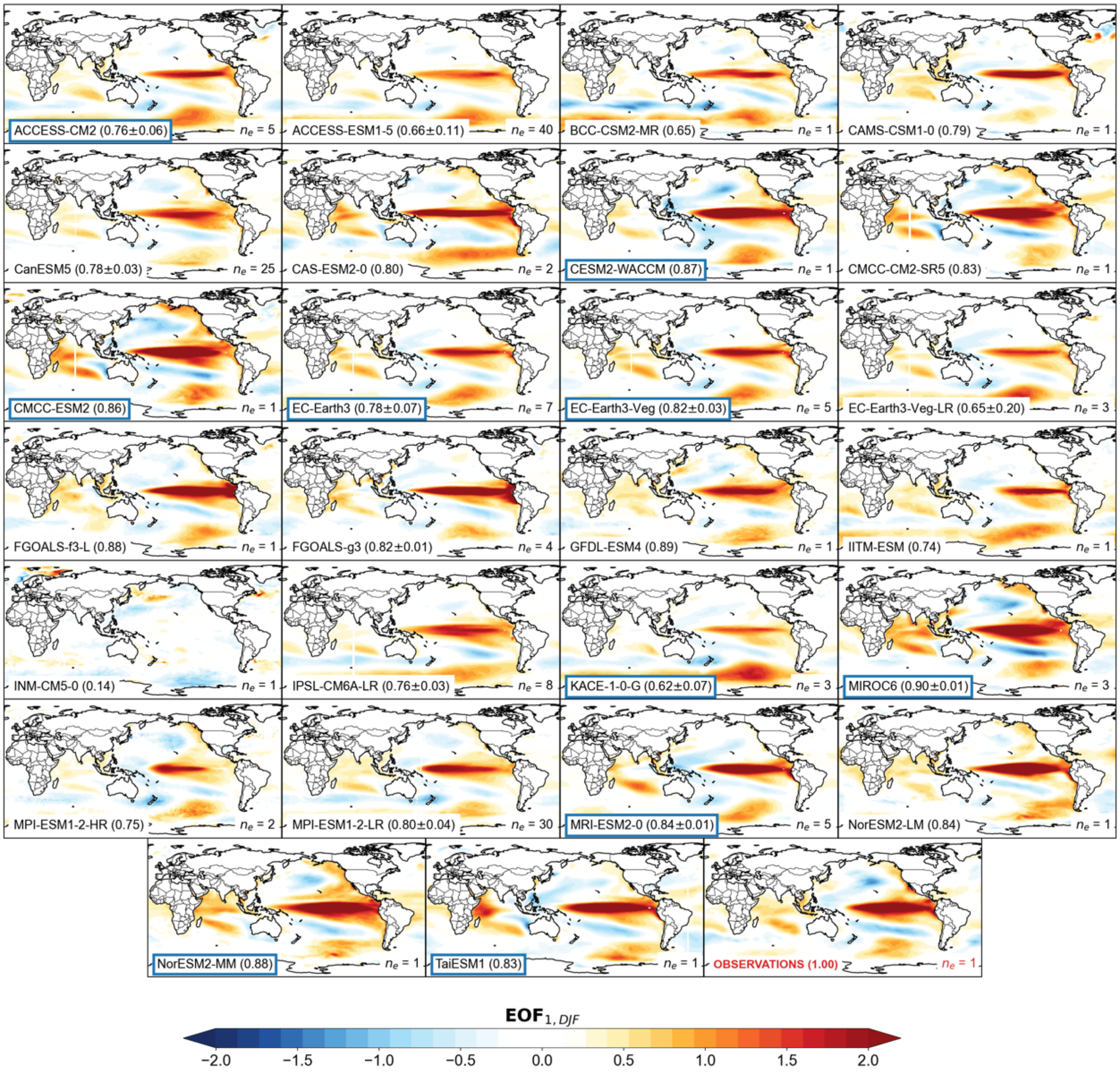

Supplementary Figure 4 | Comparison of the first principal variability pattern (EOF1) of seasonal sea surface temperature (SST) between 26 models and observations during winter (DJF) over 1964-2014. Numbers in parentheses represent the multi-ensemble mean pattern correlations of EOF1 obtained from each model and observations. n_e indicates the number of ensemble members used in each model. For models that $n_e \geq 3$, standard deviation is included. Blue boxes indicate the best 10 performing models that are selected for evaluating future changes in seasonal precipitation predictability.

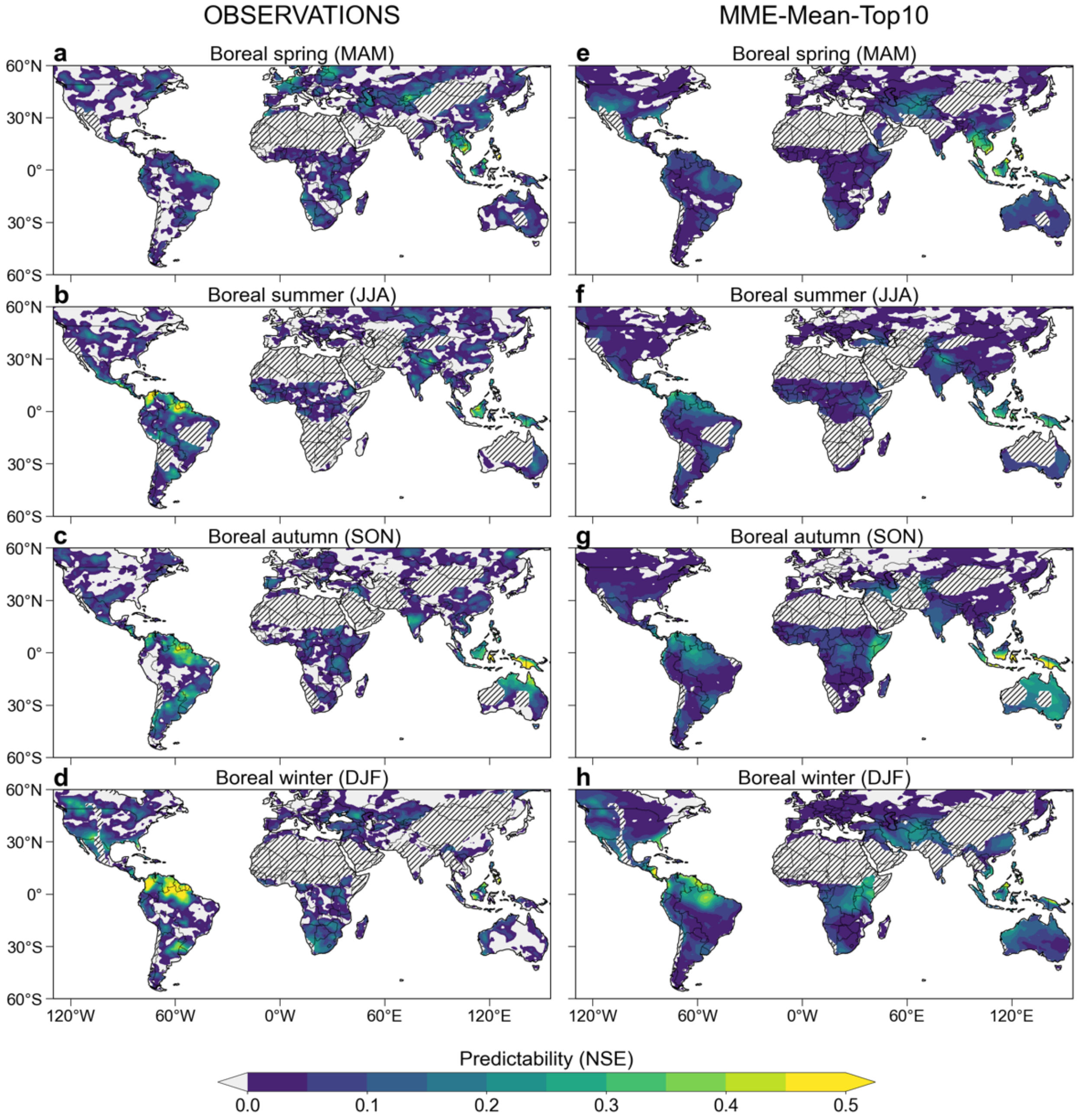

Supplementary Figure 5 | Predictability of seasonal precipitation from observations and CMIP6 models for different seasons during the historical period (1964-2014). a-d, Predictability of seasonal precipitation from observations. **e-h,** Multi-model ensemble mean predictability of seasonal precipitation from 32 ensemble members of the top 10 models (MME-Mean-Top10), identified based on their performance in capturing the spatial pattern of the observed teleconnections. Predictability is obtained from the best-2-PCs model using previous-season sea surface temperatures.

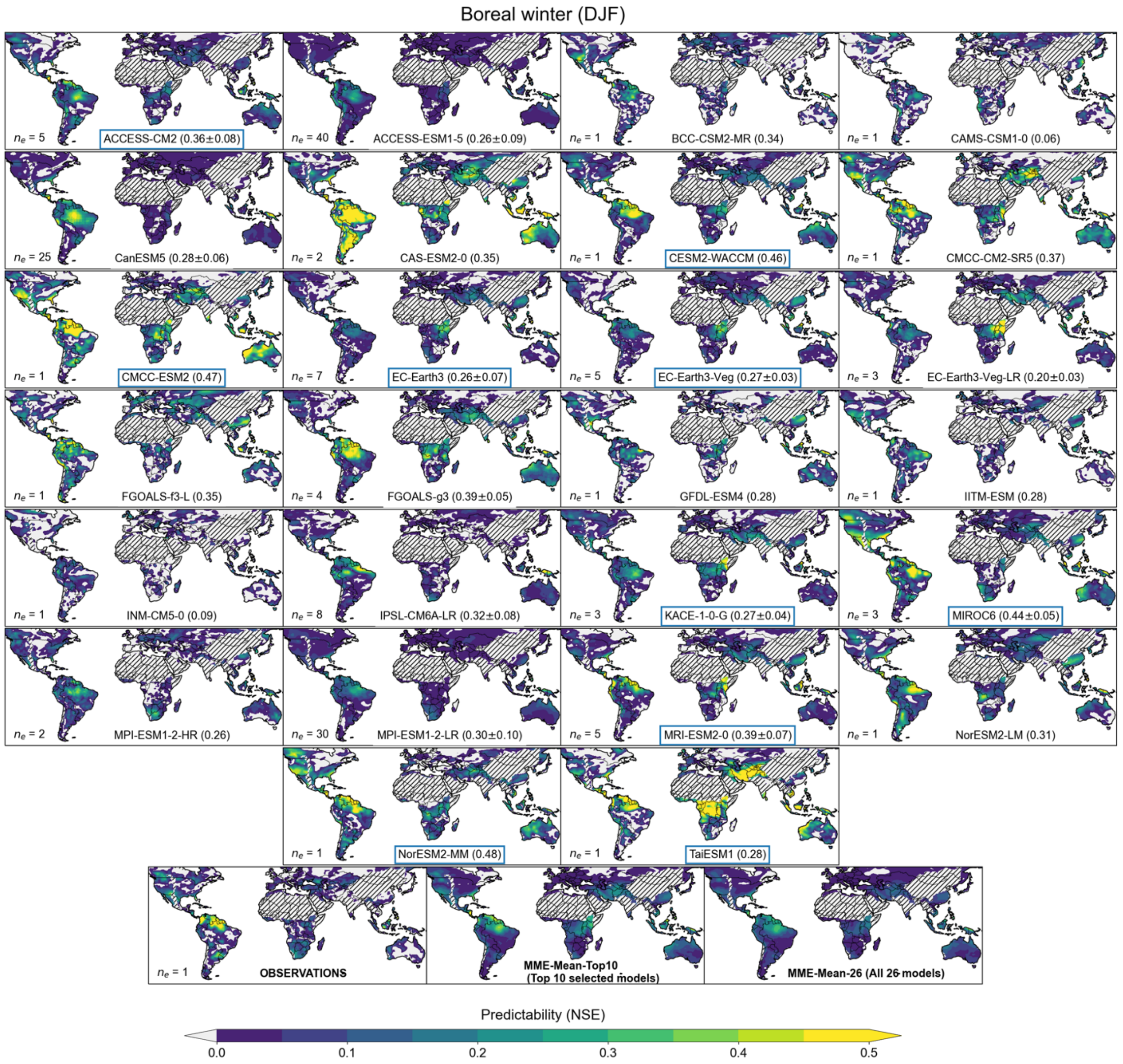

Supplementary Figure 6 | Comparison of predictability of winter precipitation (DJF) between observations and climate models during the historical period (1964-2014). Predictability is obtained from the best-2-PCs model using mean autumn (SON) sea surface temperature (SST). Numbers in paratheses represent the multi-ensemble mean $\overline{\text{PCC}}_{\text{NSE}}$ between each model and observations. n_e indicates the number of ensemble members used in each model. For models that $n_e \geq 3$, standard deviation is included. Blue boxes inside the panels indicate the best 10 performing models selected for evaluating future changes in seasonal precipitation predictability. Multi-model ensemble means of the best 10 (MME-Mean-Top10) and all 26 (MME-Mean-26) models are also included.

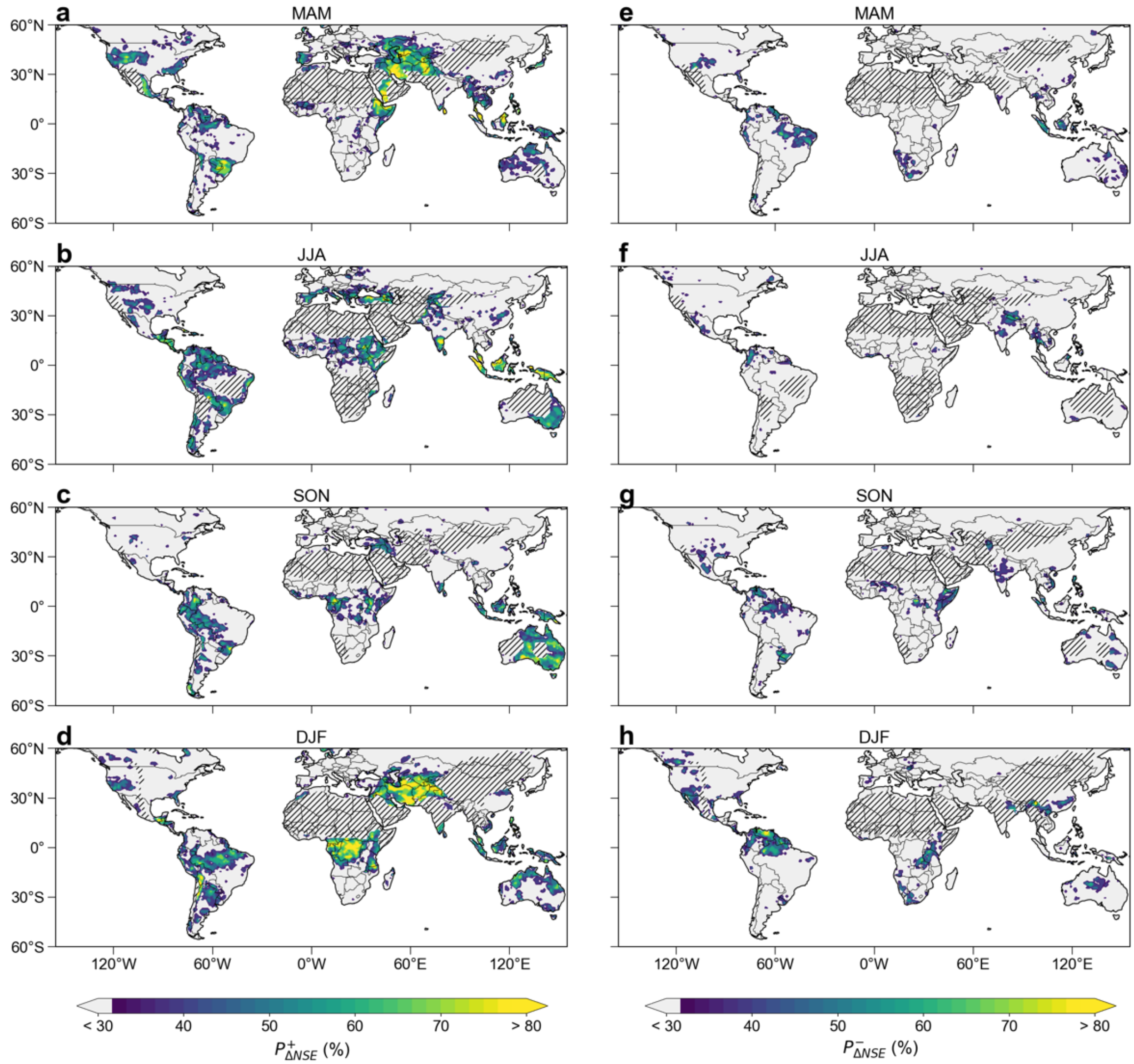

Supplementary Figure 7 | a-d, Percentage of models (%) showing increase in seasonal precipitation predictability ($\Delta NSE \geq 0.05$) for different seasons. **e-h,** Percentage of models showing decrease in seasonal precipitation predictability ($\Delta NSE \leq -0.05$) for different seasons. This analysis was performed on the set of 32 ensemble members of the 10 models shown in grey in Supplementary Table S2.

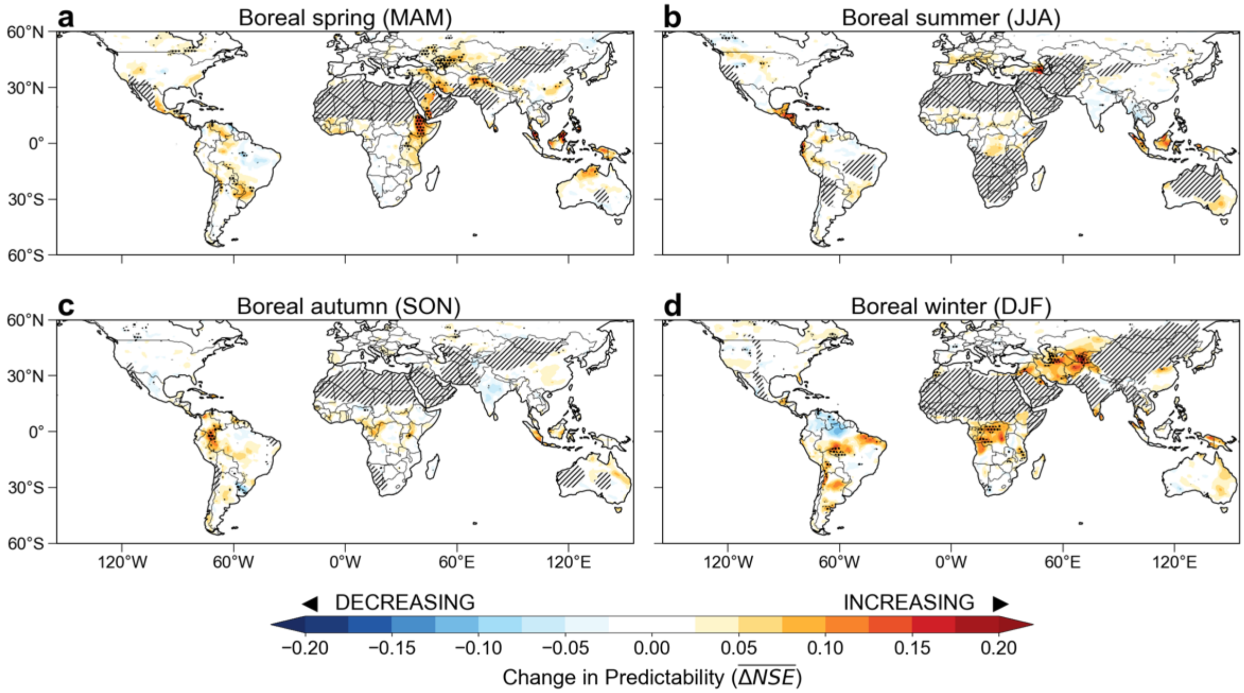

Supplementary Figure 8 | Projected changes in the predictability of seasonal precipitation for SSP2-4.5. a-d, Multi-model ensemble mean changes in seasonal precipitation predictability (ΔNSE) between historical (1964-2014) and future (2049-2099 and SSP2-4.5) periods obtained from 32 ensemble members of the top 10 selected CMIP6 models for (**a**) boreal spring, (**b**) boreal summer, (**c**) boreal autumn, and (**d**) boreal winter. Stippling indicates regions where at least 80% of the models show the same sign of change. Grey dashed regions indicate arid areas whose long-term mean total seasonal precipitation is less than 50 mm, which were excluded from the analyses.

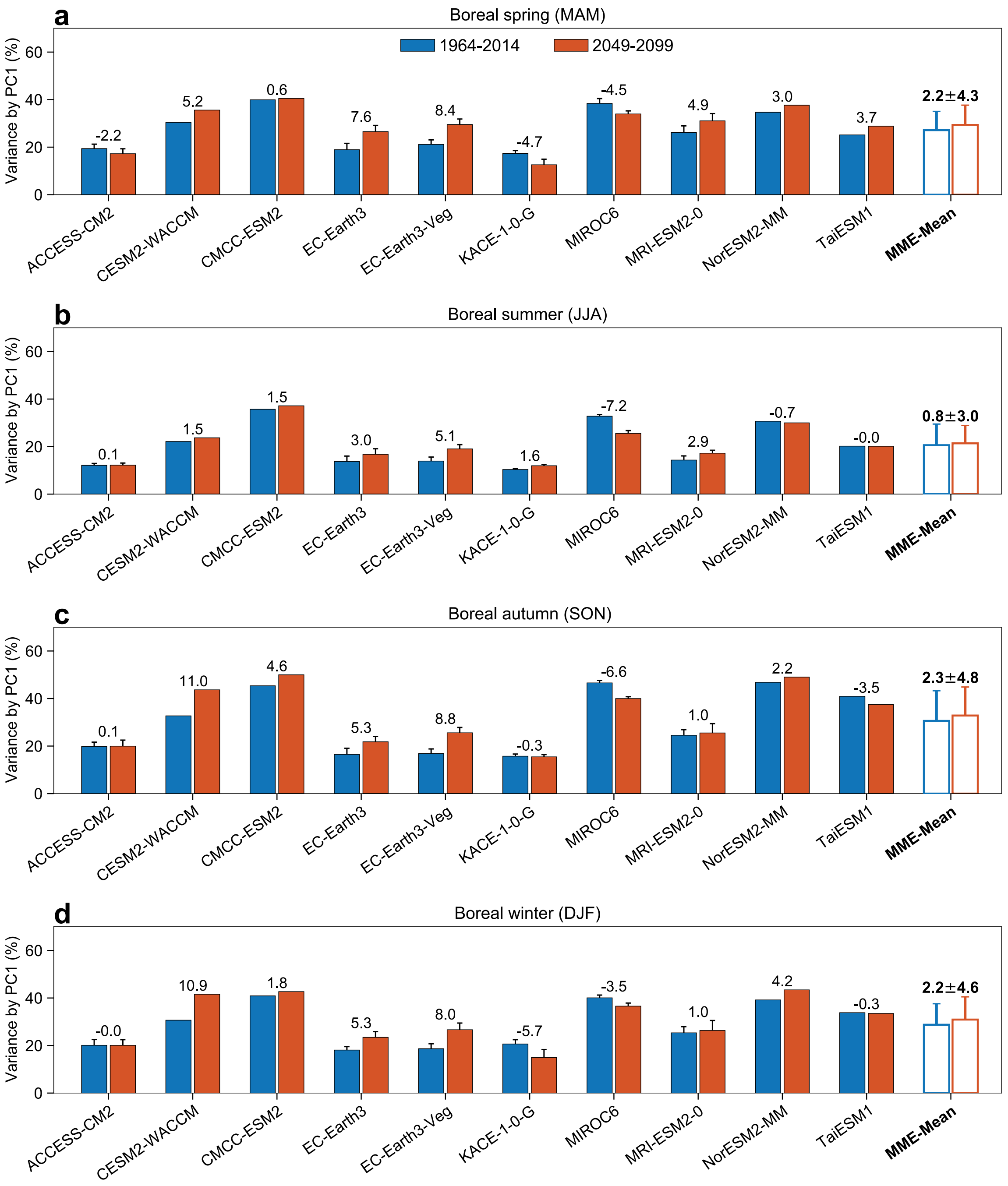

Supplementary Figure 9 | Projected changes in El Niño-Southern Oscillation (ENSO) variability for SSP3-7.0 relative to the historical period. Comparison of the variance explained by the first principal component (PC1) of sea surface temperature (SST) in the recent past (1964-2014) and future (2049-2099; SSP3-7.0) periods for 10 selected CMIP6 models during (**a**) boreal spring, (**b**) boreal summer, (**c**) boreal autumn, and (**d**) boreal winter seasons. Numbers on top of the bars represent the difference between the two periods (future – historical) for each model. Error bars at each model represent the intra-model variability (standard deviation), whereas the MME-Mean estimates represent the inter-model variability.

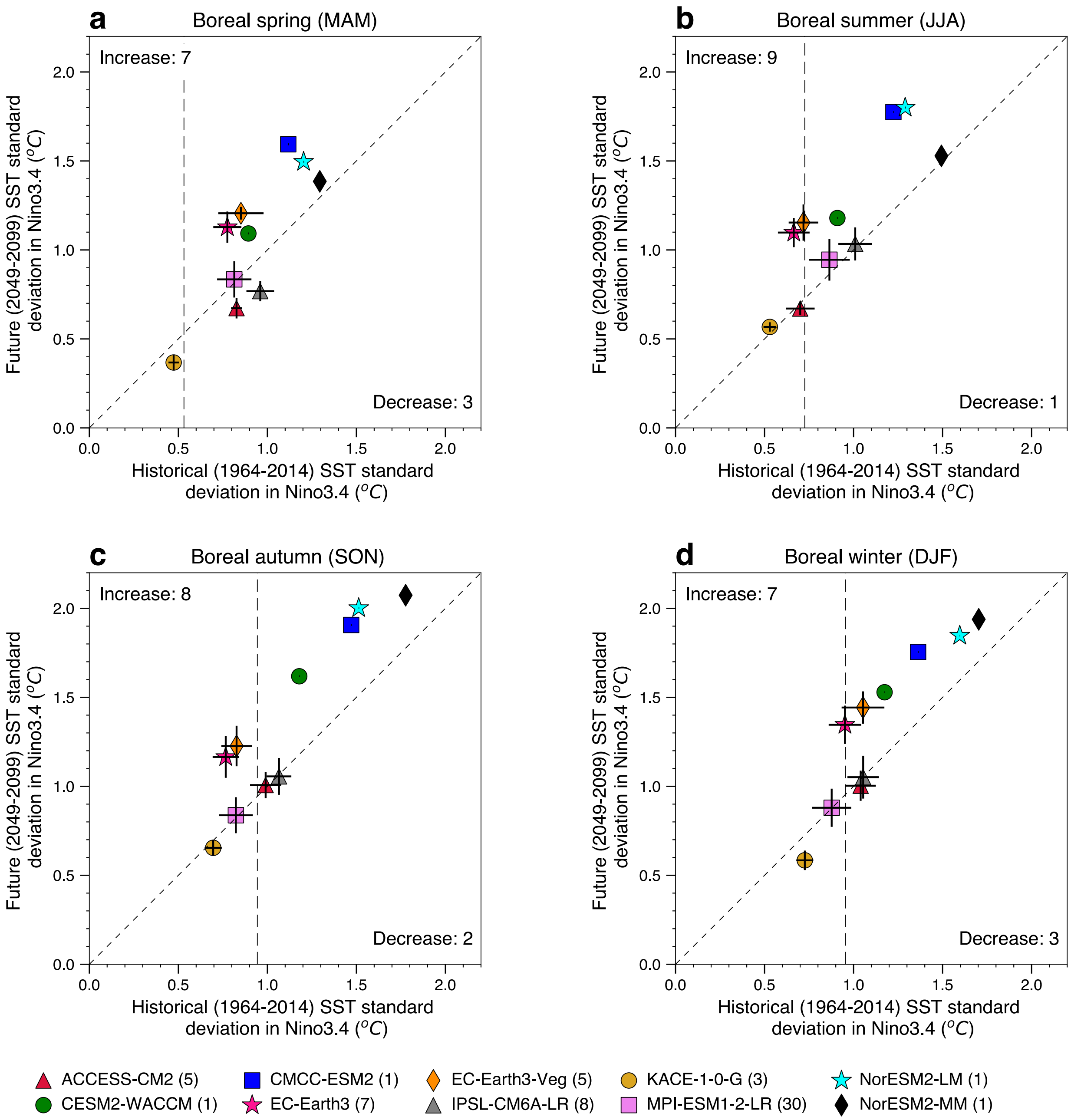

Supplementary Figure 10 | Projected changes in Niño3.4 variability for SSP3-7.0. Comparison of Niño3.4 sea surface temperature (SST) interannual standard deviation for historical (1964-2014) and future (2049-2099; SSP3-7.0) periods for the top 10 performing CMIP6 models during (**a**) boreal spring, (**b**) boreal summer, (**c**) boreal autumn, and (**d**) boreal winter seasons. The number of models generating an increase/decrease in Niño3.4 variability is indicated in the top-left/bottom-right corner. Vertical dashed lines represent the Niño3.4 SST interannual standard deviation of observations during the historical period.

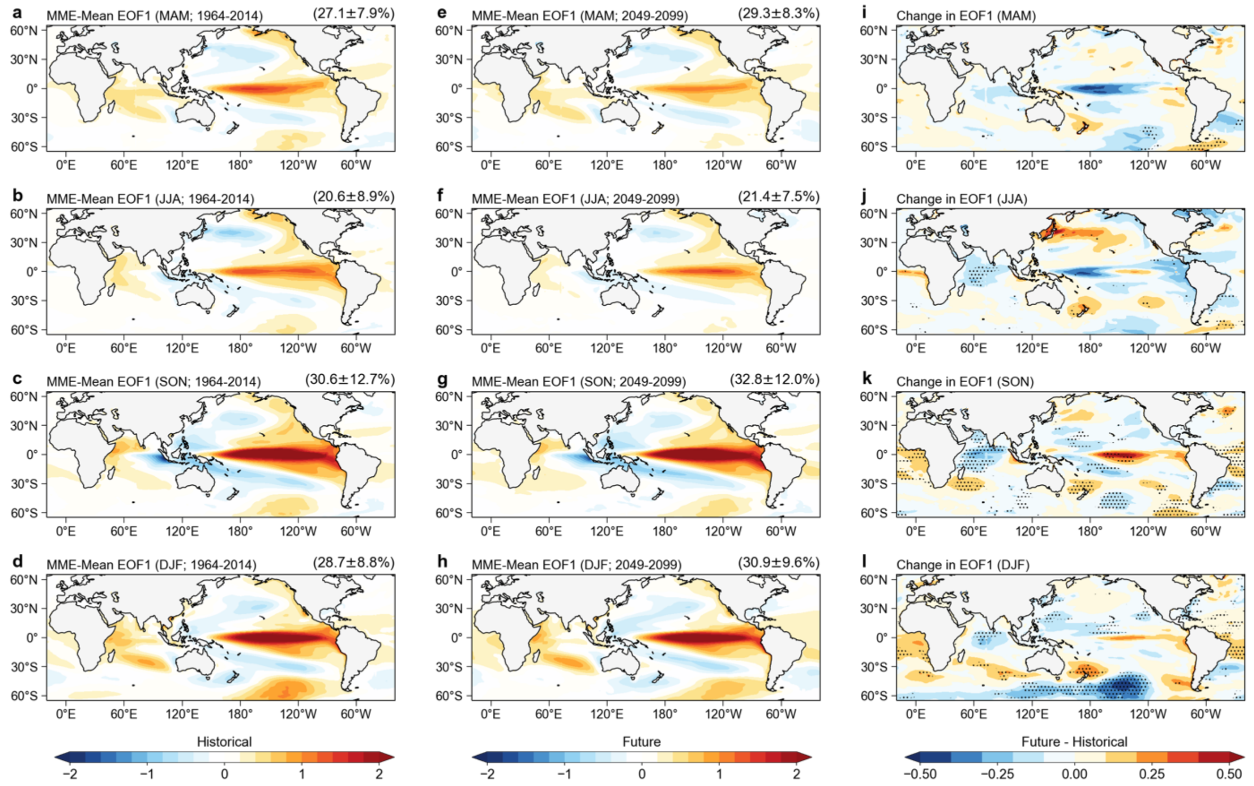

Supplementary Figure 11 | Changes in the first principal variability patterns for SSP3-7.0. a-d, First principal variability patterns (EOF1) of seasonal sea surface temperature (SST) anomalies representing El Niño-Southern Oscillation (ENSO) based on the multi-model mean of the top 10 selected CMIP6 models over the historical (1964-2014) period for (**a**) boreal spring, (**b**) boreal summer, (**c**) boreal autumn, and (**d**) boreal winter. **e-h**, Same as in **a-d** but for future (2049-2099; SSP3-7.0) period. **i-l,** Change in the first principal variability patterns ($\overline{\Delta \mathrm{EOF1}}$) between future (2049-2099) and historical (1964-2014) periods based on the multi-model mean of the top 10 selected CMIP6 models for (**i**) boreal spring, (**j**) boreal summer, (**k**) boreal autumn, and (**l**) boreal winter. Numbers in parentheses on the top-right corner in (**a-h**) show the multi-model ensemble (MME) mean ± standard deviation of the variance explained by the first principal component. Stippling in (**i-l**) indicates regions where at least 80% of the models show the same sign of change.

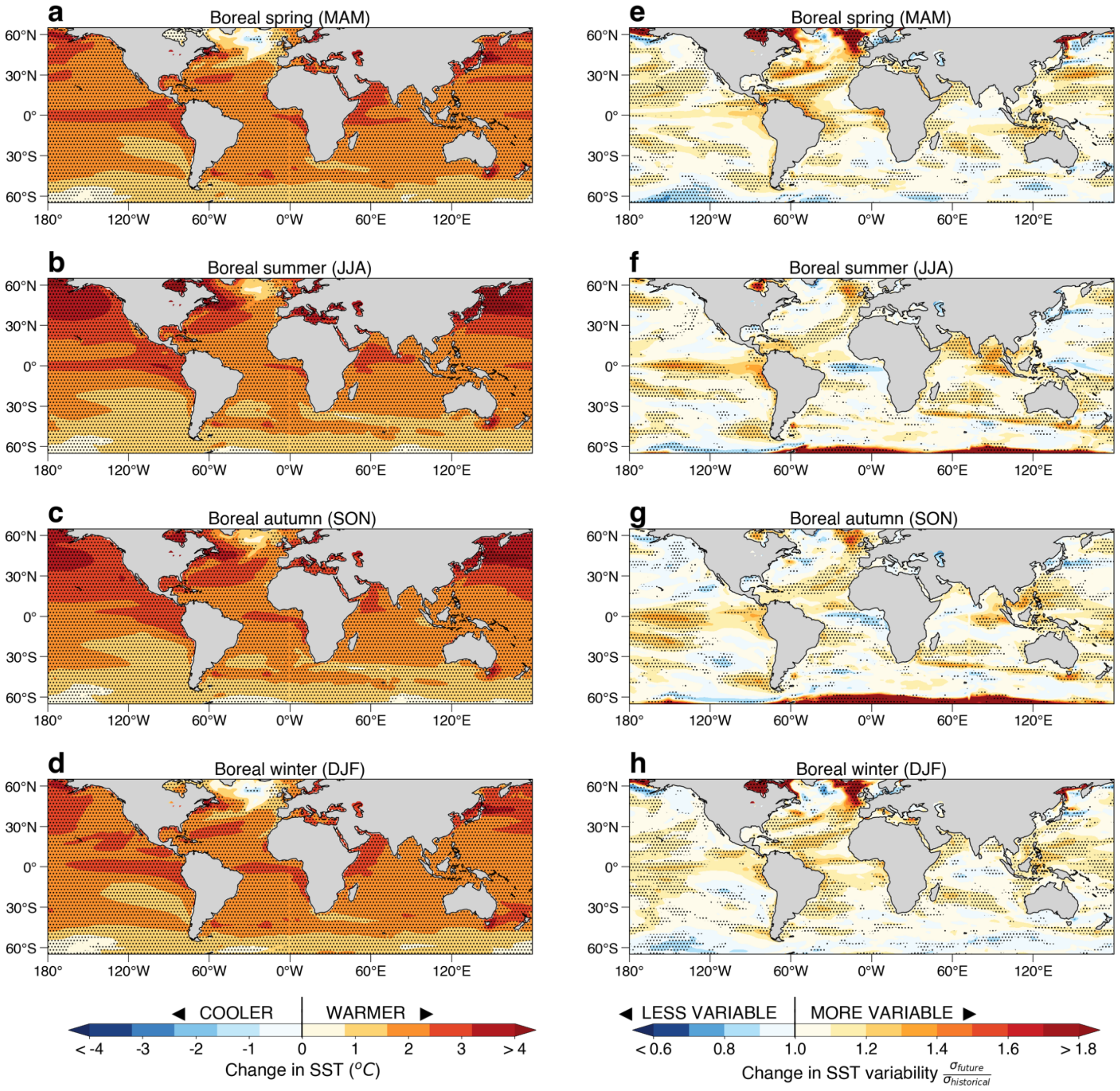

Supplementary Figure 12 | Projected changes in magnitude and year-to-year variability of seasonal sea surface temperature (SST) for SSP3-7.0. a-d, Projected changes in seasonal SST between historical (1964-2014) and future (2049-2099; SSP3-7.0) periods during (**a**) boreal spring, (**b**) boreal summer, (**c**) boreal autumn, and (**d**) boreal winter. **e-h,** Projected changes in standard deviation of seasonal SST between historical and future (SSP3-7.0) periods ($\sigma_{\text{future}}/\sigma_{\text{historical}}$) during (**e**) boreal spring, (**f**) boreal summer, (**g**) boreal autumn, and (**h**) boreal winter. Stippling indicates regions where at least 80% of the models show the same sign of change. This set of projected changes was computed for the set of 32 ensemble members of the 10 models shown in grey in Supplementary Table S2.

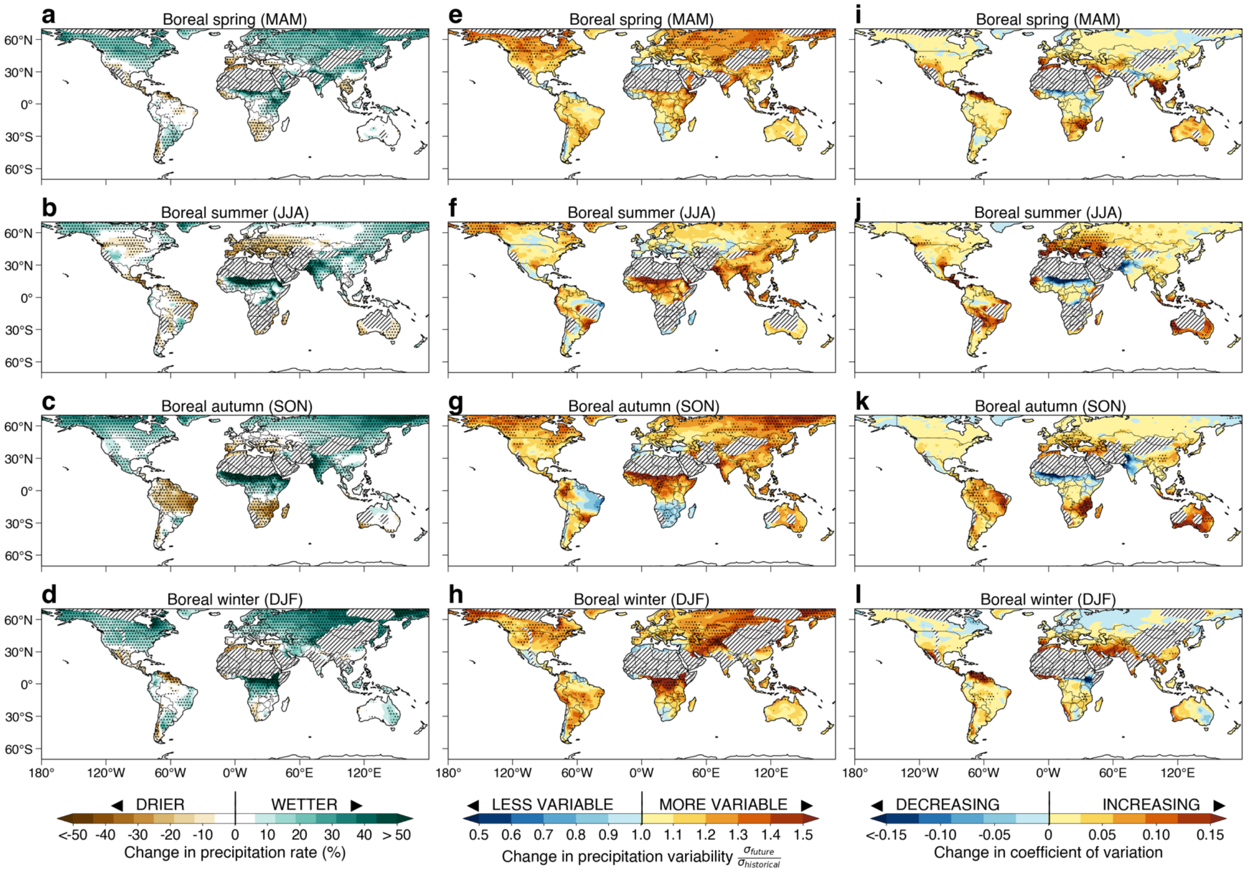

Supplementary Figure 13 | Projected changes in seasonal total precipitation for SSP3-7.0. a-d, Projected percent changes in seasonal total precipitation between historical (1964-2014) and future (2049-2099; SSP3-7.0) periods during (**a**) boreal spring, (**b**) boreal summer, (**c**) boreal autumn, and (**d**) boreal winter. **e-h,** Same as **a-d** but for change in precipitation variability represented by the ratio of the standard deviation of seasonal precipitation between future (and historical ($\sigma_{\text{future}}/\sigma_{\text{historical}}$) periods. **i-l**, Same as **a-d** but for change in the coefficient of variation between future and historical ($\text{CV}_{\text{future}} - \text{CV}_{\text{historical}}$) periods. Stippling indicates regions where at least 80% of the models show the same sign of change. Grey dashed regions indicate desert areas with mean observed seasonal precipitation P_{season}<50 mm, which were excluded from the analyses. This set of projected changes was computed for the set of 32 ensemble members of the 10 models shown in grey in Supplementary Table S2.

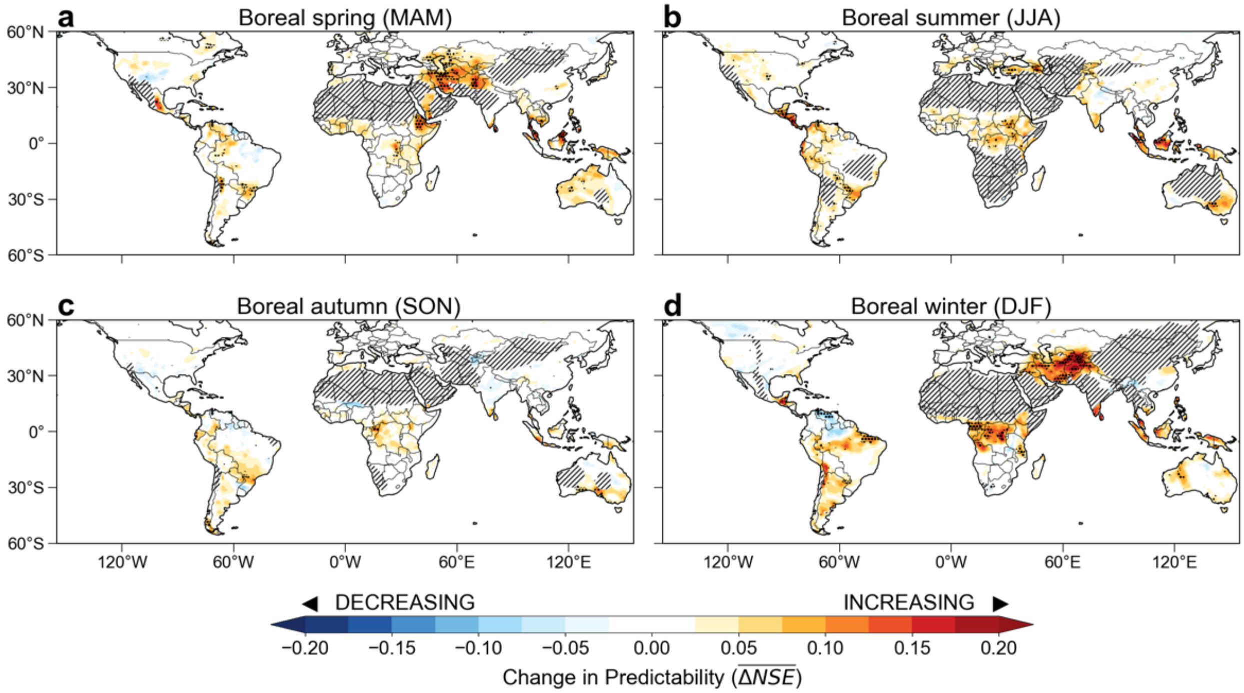

Supplementary Figure 14 | Same as Figure 4 in the main text (under SSP3-7.0) but using linear detrending method.

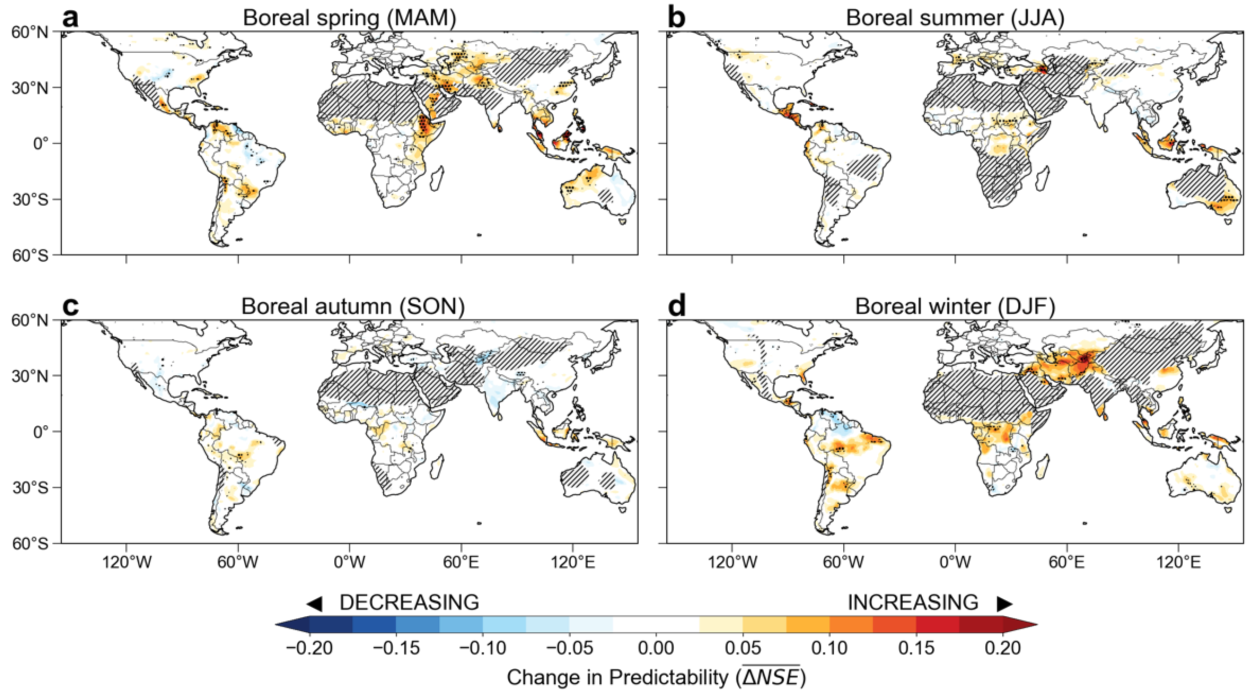

Supplementary Figure 15 | Same as Supplementary Figure 8 (under SSP2-4.5) but using linear detrending method.

Supplementary References

1 Bi, D. *et al.* Configuration and spin-up of ACCESS-CM2, the new generation Australian Community Climate and Earth System Simulator Coupled Model. *Journal of Southern Hemisphere Earth Systems Science* **70**, 225-251 (2020).
2 Ziehn, T. *et al.* The Australian Earth System Model: ACCESS-ESM1.5. *Journal of Southern Hemisphere Earth Systems Science* **70**, 193-214 (2020).
3 Wu, T. *et al.* BCC-CSM2-HR: a high-resolution version of the Beijing Climate Center Climate System Model. *Geosci. Model Dev.* **14**, 2977-3006 (2021). https://doi.org:10.5194/gmd-14-2977-2021
4 Xin-Yao Rong, J. L. I. H.-M. C. Y.-F. X. I. N. J.-Z. S. U. L.-J. H. U. A. Z.-Q. Z. Introduction of CAMS-CSM model and its participation in CMIP6. *Advances in Climate Change Research* **15**, 540-544 (2019).
5 Swart, N. C. *et al.* The Canadian Earth System Model version 5 (CanESM5.0.3). *Geosci. Model Dev.* **12**, 4823-4873 (2019). https://doi.org:10.5194/gmd-12-4823-2019
6 Zhang, H. *et al.* Description and Climate Simulation Performance of CAS-ESM Version 2. *Journal of Advances in Modeling Earth Systems* **12**, e2020MS002210 (2020). https://doi.org:https://doi.org/10.1029/2020MS002210
7 Danabasoglu, G. *et al.* The Community Earth System Model Version 2 (CESM2). *Journal of Advances in Modeling Earth Systems* **12**, e2019MS001916 (2020). https://doi.org:https://doi.org/10.1029/2019MS001916
8 Cherchi, A. *et al.* Global Mean Climate and Main Patterns of Variability in the CMCC-CM2 Coupled Model. *Journal of Advances in Modeling Earth Systems* **11**, 185-209 (2019). https://doi.org:https://doi.org/10.1029/2018MS001369
9 Lovato, T. *et al.* CMIP6 Simulations With the CMCC Earth System Model (CMCC-ESM2). *Journal of Advances in Modeling Earth Systems* **14**, e2021MS002814 (2022). https://doi.org:https://doi.org/10.1029/2021MS002814
10 Döscher, R. *et al.* The EC-Earth3 Earth system model for the Coupled Model Intercomparison Project 6. *Geosci. Model Dev.* **15**, 2973-3020 (2022). https://doi.org:10.5194/gmd-15-2973-2022
11 He, B. *et al.* CAS FGOALS-f3-L model dataset descriptions for CMIP6 DECK experiments. *Atmospheric and Oceanic Science Letters* **13**, 582-588 (2020). https://doi.org:10.1080/16742834.2020.1778419
12 Li, L. *et al.* The Flexible Global Ocean-Atmosphere-Land System Model Grid-Point Version 3 (FGOALS-g3): Description and Evaluation. *Journal of Advances in Modeling Earth Systems* **12**, e2019MS002012 (2020). https://doi.org:https://doi.org/10.1029/2019MS002012
13 Dunne, J. P. *et al.* The GFDL Earth System Model Version 4.1 (GFDL-ESM 4.1): Overall Coupled Model Description and Simulation Characteristics. *Journal of Advances in Modeling Earth Systems* **12**, e2019MS002015 (2020). https://doi.org:https://doi.org/10.1029/2019MS002015
14 Swapna, P. *et al.* Long-Term Climate Simulations Using the IITM Earth System Model (IITM-ESMv2) With Focus on the South Asian Monsoon. *Journal of Advances in Modeling Earth Systems* **10**, 1127-1149 (2018). https://doi.org:https://doi.org/10.1029/2017MS001262
15 Volodin, E. & Gritsun, A. Simulation of observed climate changes in 1850–2014 with climate model INM-CM5. *Earth Syst. Dynam.* **9**, 1235-1242 (2018). https://doi.org:10.5194/esd-9-1235-2018
16 Boucher, O. *et al.* Presentation and Evaluation of the IPSL-CM6A-LR Climate Model. *Journal of Advances in Modeling Earth Systems* **12**, e2019MS002010 (2020). https://doi.org:https://doi.org/10.1029/2019MS002010
17 Lee, J. *et al.* Evaluation of the Korea Meteorological Administration Advanced Community Earth-System model (K-ACE). *Asia-Pacific Journal of Atmospheric Sciences* **56**, 381-395 (2020). https://doi.org:10.1007/s13143-019-00144-7
18 Tatebe, H. *et al.* Description and basic evaluation of simulated mean state, internal variability, and climate sensitivity in MIROC6. *Geosci. Model Dev.* **12**, 2727-2765 (2019). https://doi.org:10.5194/gmd-12-2727-2019

19 Gutjahr, O. *et al.* Max Planck Institute Earth System Model (MPI-ESM1.2) for the High-Resolution Model Intercomparison Project (HighResMIP). *Geosci. Model Dev.* **12**, 3241-3281 (2019). https://doi.org:10.5194/gmd-12-3241-2019

20 Mauritsen, T. *et al.* Developments in the MPI-M Earth System Model version 1.2 (MPI-ESM1.2) and Its Response to Increasing CO2. *Journal of Advances in Modeling Earth Systems* **11**, 998-1038 (2019). https://doi.org:https://doi.org/10.1029/2018MS001400

21 Yukimoto, S. *et al.* The Meteorological Research Institute Earth System Model Version 2.0, MRI-ESM2.0: Description and Basic Evaluation of the Physical Component. *Journal of the Meteorological Society of Japan. Ser. II* **97**, 931-965 (2019). https://doi.org:10.2151/jmsj.2019-051

22 Seland, Ø. *et al.* Overview of the Norwegian Earth System Model (NorESM2) and key climate response of CMIP6 DECK, historical, and scenario simulations. *Geosci. Model Dev.* **13**, 6165-6200 (2020). https://doi.org:10.5194/gmd-13-6165-2020

23 Lee, W. L. *et al.* Taiwan Earth System Model Version 1: description and evaluation of mean state. *Geosci. Model Dev.* **13**, 3887-3904 (2020). https://doi.org:10.5194/gmd-13-3887-2020
